# Suppression of NLRP3 inflammasome by a small molecule targeting CK1α–β-catenin–NF-κB and CK1α–NRF2–mitochondrial OXPHOS pathways during mycobacterial infection

**DOI:** 10.3389/fimmu.2025.1553093

**Published:** 2025-02-28

**Authors:** Qing Guan, Huan Xiong, Xiangyu Song, Sheng Liu, Yuanjun Guang, Qi Nie, Yan Xie, Xiao-Lian Zhang

**Affiliations:** ^1^ State Key Laboratory of Virology and Biosafety, Hubei Province Key Laboratory of Allergy and Immunology, Department of Immunology Wuhan University Taikang Medical School (School of Basic Medical Sciences) Wuhan University, Wuhan, China; ^2^ Frontier Science Center for Immunology and Metabolism, Department of Allergy Zhongnan Hospital, Wuhan University School of Medicine, Wuhan, China; ^3^ Department of Laboratory Medicine, Third Affiliated Hospital of Sun Yat-sen University, Guangzhou, China; ^4^ Department of Multidrug-Resistant and Rifampicin-Resistant Tuberculosis (MDR/RR-TB), Wuhan Jinyintan Hospital, Tongji Medical College of Huazhong University of Science and Technology, Wuhan, Hubei, China; ^5^ College of Life Sciences and Health, Wuhan University of Science and Technology, Wuhan, Hubei, China

**Keywords:** pyrvinium pamoate, mycobacterium tuberculosis, NLRP3 inflammasome, GSDMD, pyroptosis, CK1α-β-catenin-NF-κB pathway, CK1α-NRF2-mitochondrial OXPHOS pathway

## Abstract

**Introduction:**

Pyroptosis is an important inflammatory form of cell death and *Mycobacterium tuberculosis* (*M.tb*) chronic infection triggers excessive inflammatory pyroptosis of macrophages. Our previous research has confirmed that a small compound pyrvinium pamoate (PP) could inhibit inflammatory pathological changes and mycobacterial burden in *M.tb*-infected mice, but the potential mechanism of PP for inhibiting *M.tb*-induced inflammation remains unexplored.

**Methods:**

The effects of PP on the NLRP3-ASC-Casp1 inflammasome assembly and activation, gasdermin D (GSDMD) mediated pyroptosis and inflammatory cytokines expression were assessed in human THP-1-derived macrophages after *M.tb* H37Rv/H37Ra/ *Salmonella typhimurium* (*S. typhimurium*) infection or LPS treatment by Transcriptome sequencing, RT-qPCR, Co-immunoprecipitation and Western Blot (WB) analysis. The lactate dehydrogenase (LDH) release assay was used to evaluate the CC50 of PP in *M.tb*-infected THP-1 cells.

**Results:**

We found that *M.tb*/*S. typhimurium* infection and LPS treatment significantly activate NLRP3-ASC-Casp1 inflammasome activation, GSDMD-mediated pyroptosis and inflammatory cytokines (IL-1β and IL-18) expression in macrophages, whereas PP could suppress these inflammatory effects in a dose dependent manner. Regarding the PP-inhibition mechanism, we further found that this inhibitory activity is mediated through the PP-targeting casein kinase 1A1 (CK1α)–β-catenin–NF-κB pathway and CK1α–NRF2–mitochondrial oxidative phosphorylation (OXPHOS) pathway. In addition, a CK1α specific inhibitor D4476 or CK1α siRNA could reverse these inhibitory effects of PP on bacteria-induced inflammatory responses in macrophages.

**Conclusions:**

This study reveals a previously unreported mechanism that pyrvinium can inhibit NLRP3 inflammasome and GSDMD–IL-1β inflammatory pyroptosis via targeting suppressing CK1α–β-catenin–NF-κB and CK1α–NRF2–mitochondrial OXPHOS pathways, suggesting that pyrvinium pamoate holds great promise as a host directed therapy (HDT) drug for mycobacterial-induced excessive inflammatory response.

## 1 Introduction

Tuberculosis (TB) is a preventable and usually curable disease caused by *Mycobacterium tuberculosis* (*M.tb*), which affects more than 10.8 million people and kills 1.25 million people in 2023 according to Global Tuberculosis Report 2024 (1). The balance of host immune responses affects the outcome and the pathology of TB. *M.tb* infection is usually accompanied by intense host local excessive inflammatory response, which is critical for its pathogenesis and for restricting bacterial growth (2–4). The inflammatory response is a two-edged sword in TB pathogenesis. While it could control the infection, the excessive inflammatory response can lead to tissue damage, organ failure or even death (5–8). Host directed therapy (HDT) is a novel anti-TB adjuvant therapy strategy that has been proposed in recent years. HDT aims to regulate host immune response and reduce excessive inflammatory response and pathological damage, thereby improving clinical treatment outcomes (9).

Macrophages act as the primary host cell for *M.tb*, and secret large amount of pro-inflammatory cytokines such as IL-1β, IL-6 and TNF-α for eliminating intracellular *M.tb* and eliciting excessive inflammatory response. Inflammasomes play a crucial role in immunity by mediating the release of inflammatory cytokines mainly from macrophages (10). To date, five different types of inflammasome have been identified according to their core pattern recognition receptor (PRR)s, namely nucleotide-binding oligomerization domain-like receptor (NLR)P1, NLRP3, NLRC4, absent in melanoma 2-like receptor (AIM2) and Pyrin (11–13), among which the most thoroughly studied inflammasome is NLRP3 inflammasome. NLRP3 inflammasome activation requires a second signal delivering from potassium efflux, calcium influx, ROS production, or lysosomal rupture (14, 15). The canonical NLRP3 inflammasome complex is an intracellular protein complex consisting of the sensor NLRP3, the adaptor ASC (apoptosis-associated speck-like proteins that contain a caspase recruitment domain), and pro-caspase-1 (pro-Casp1). After sensing PAMP (pathogen-associated molecular pattern) or DAMP (damage-associated molecular pattern) signals, presumably via the leucine-rich repeat (LRR) domain of NLRP3, NLRP3 monomers induce oligomerize and interact with the pyrin domain (PYD) domain of ASC through homophilic interactions. The adaptor ASC then recruits the cysteine protease pro-caspase-1 via a caspase recruitment domain (CARD) (15, 16). The resulting inflammasome components then form a platform on which caspase-1 is activated. Caspase-1 processes premature IL-1 family cytokines (pro-IL-1β and pro-IL-18) and cleaves gasdermin D (GSDMD). The cleaved N-terminal fragments of GSDMD, in turn, forms pores in membranes to allow release of IL-1β, IL-18 and other inflammatory molecules and lysis of cell, i.e. pyroptosis (17). Pyrvinium pamoate (PP) acts as a small classical anthelminthic drug officially approved by the US Food and Drug Administration (US FDA). PP has also been confirmed as a Wnt/β-catenin pathway inhibitor in numerous tumor research (18). Recent research has indicated that PP has anti-inflammatory effect (19–21). Our previous research has confirmed that PP could significantly decreases mycobactericidal burden and the inflammatory pathological changes in lung, spleen and liver tissues in *M.tb*-infected mice (21). However, the mechanism of PP for inhibiting *M.tb*-induced inflammation remains unexplored.

In the present study, we demonstrate that PP could suppress *M.tb/Salmonella typhimurium (S. typhimurium) infection-* and lipopolysaccharide (LPS)-induced NLRP3**–**ASC**–**Casp-1 inflammasome activation and GSDMD**–**IL-1β inflammatory pyroptosis through suppressing casein kinase 1A1 (CK1α)–β-catenin/NF-κB signal pathway and CK1α–NRF2–mitochondrial oxidative phosphorylation (OXPHOS) pathway. While a CK1α specific inhibitor D4476 or CK1α siRNA could reverse this inhibitory effect of PP on bacteria*-*induced inflammatory responses. Our findings offer robust evidence that PP can inhibit NLRP3 inflammasome activation and GSDMD**–**IL-1β inflammatory pyroptosis, and PP holds great promise as a HDT drug for bacterial-induced inflammatory pyroptosis.

## 2 Materials and methods

### 2.1 Bacterial strains and cultures

*M.tb* H37Rv/H37Ra [strain American Type Culture Collection (ATCC) 27294] was maintained on Lowenstein-Jensen medium. The mycobacterial strains were grown in Middlebrook 7H9 broth (7H9) supplemented with 10% oleic acid–albumin–dextrose–catalase (OADC, BD Biosciences, NJ, USA) and 0.05% Tween 80 (Sigma-Aldrich, Germany) or on Middlebrook 7H10 agar (BD Biosciences) supplemented with 10% OADC. *S. typhimurium* C5 strain was maintained in our laboratory and grown in Lysogeny broth (LB) Medium.

### 2.2 Cell culture and treatment

The human myelomonocytic cell line THP-1 was cultured in RPMI 1640 medium (Gibco, USA), supplemented with 10% fetal bovine serum (FBS). THP-1 cells were differentiated into macrophages by treatment with 100 ng/mL phorbol myristate acetate (PMA) for 24 h. Then H37Ra, H37Rv, *S. typhimurium* C5 or LPS (Sigma-Aldrich, Cat. No. L2630) were respectively used to infect/stimulate THP-1 cell for 4 h, the cells were washed with PBS for 3 times, and then PP (MCE, HY-A0293, MedChemExpress, USA) and/or D4476 (CK1α inhibitor, MCE, HY-10324, MedChemExpress, USA)/MSAB (β-catenin inhibitor, MCE, HY-120697)/PDTC (NF-κB inhibitor, MCE, HY-18738)/NRF2-IN-1 (NRF2 inhibitor, MCE, HY-101025) was added and cultured for another 24 h.

THP-1-derived macrophages were transiently transfected with CK1α siRNA synthesized by Sangon Biotech (Shanghai, China) for 48 h using the transfection reagent CALNP RNAi (D-Nano Therapeutics, DN001-10, Beijing, China). The siRNA sequences were listed in **Supplementary Table 2**. The cell lysates were used for analysis of CK1α protein expression.

### 2.3 Transcriptome sequencing

THP-1 cells were seeded in 6-well plates at a density of 1.2 × 10^6^ cells and treated with 100 ng/mL PMA for 24 h. Cells were infected with H37Rv at an MOI of 10 for 4 h, then cells were washed three times with PBS to remove unphagocytosed bacteria and fresh medium containing 3 µg/mL PP was added. Plates were incubated at 37°C in 5% CO_2_ for 24 h, then cells were washed three times with PBS and 1 mL TRIzol reagent was added to lyse cells. Finally, the lysis solution was collected for Transcriptome Sequencing in Biozeron.

### 2.4 Reverse transcription–quantitative realtime polymerase chain reaction (RT-qPCR)

To detect the mRNA expression of NLRP1, NLRP3, NLRC4, AIM2 and Pyrin in THP-1 cells after infecting with H37Ra, THP-1 cells were seeded in in 12-well plates at a density of 5 × 10^5^ cells and treated with 100 ng/mL PMA for 24 h, cells were then harvested for RNA analysis at indicated time points after infecting with H37Ra at an MOI of 10.

To detect the inhibition effect of PP on the mRNA expression of NLRP3, GSDMD, pro-IL-1β, IL-18 and mitochondrial OXPHOS related genes in H37Ra/H37Rv infected THP-1 cells, THP-1 cells were seeded in 12-well plates at a density of 5 × 10^5^ cells and treated with 100 ng/mL PMA for 24 h. Cells were infected with H37Rv/H37Ra at an MOI of 10 for 4 h, then cells were washed three times with PBS to remove unphagocytosed bacteria and fresh medium containing PP or DMSO was added. Plates were incubated at 37°C in 5% CO_2_ for 24 h. Total RNA was extracted from the cells using TRIzol reagent (Life Technologies Corporation, USA).

The RT-qPCR reactions were run on Quantagene q225MX using the standard cycling conditions. Target gene expression levels were normalized based on glyceraldehyde-3-phosphate dehydrogenase (GAPDH). The primer sequences of individual genes were shown in **Supplementary Table 1**. Relative RNA levels were calculated by the comparative cycle threshold (CT) method (2^−ΔΔCT^ method), where CT indicates the amplification cycle number at which the fluorescence generated within a reaction rises above a defined threshold fluorescence, and ΔΔCT = experimental sample (Ct_target gene_ − Ct_GAPDH_) − control sample (Ct_target gene_ − Ct_GAPDH_).

### 2.5 Immunoprecipitation

THP-1 cells were seeded in 6 cm plate at a density of 5 × 10^6^ cells and treated with 100 ng/mL PMA for 24 h. Cells were infected with or without H37Ra/H37Rv at an MOI of 10 for 4 h, then cells were washed three times with PBS to remove unphagocytosed bacteria and fresh medium containing PP or DMSO was added. Plates were incubated at 37°C in 5% CO_2_ for 24 h. Cells were harvested and then lysed in RIPA with protease inhibitor phenyl methyl sulfonyl fluoride (PMSF). The cell lysates were then incubated with anti-NLRP3 monoclonal antibody (mAb) at 4°C for 8 h. The ASC/NLRP3/caspase-1 complexes were captured using protein A+G magnetic beads and then washed three times with TBST to remove nonspecific binding protein. Elution buffer (0.1 to 0.2 M glycine and 0.1 to 0.5% Triton X-100) was used to elute the antigen. The eluted proteins were subjected to WB analysis with anti-ASC/NLRP3/caspase-1 mAbs.

### 2.6 Western blotting (WB) analysis

For WB analysis, the protein samples were isolated through SDS-PAGE, followed by transfer to the PVDF (Millipore, Billerica, MA, USA). Afterwards, the PVDF were blocked using 5% skim milk for 2 h under room temperature, incubated using appropriate primary antibodies (1:1000) under the temperature of 4°C overnight, and then incubated using the secondary peroxidase-conjugated goat anti-rabbit/mouse antibody (1:10000) for 1 h under 37°C. Finally, the Immobilon WesternBright ECL HRP substrate (K-12045-D10, Advansta, USA) was used to analyze the immunoblots. The following primary antibodies were used: anti-NLRP3 (CST, 15101, Cell Signaling Technology, USA), anti-IL-1β (ABclonal, A16288, ABclonal Biotechnology Co., Ltd., China), anti-p-NF-κB P65 (CST, 3033s), anti-ASC (CST, 67824), anti-caspase-1 (ABclonal, A0964), anti-β-actin (ABclonal, AC026), anti-GSDMD polyclonal antibody (Proteintech, 20770-1-AP), anti-Cleaved Gasdermin D (N Terminal) Rabbit mAb (ABclonal, A24059), anti-Casein Kinase 1 alpha (CK1α) (ABclonal, A9308), anti-IL-18 Rabbit pAb (ABclonal, A1115), anti-β-catenin Rabbit mAb (ABclonal, A19657), anti-NRF2 pAb (Proteintech, 16396-1-AP, Proteintech Group, Inc., China), goat anti-rabbit/mouse antibody (ABclonal, AS014, AS003). Human oxidative phosphorylation immunoblotting kit (Proteintech, PK30006) was used to assess the relative levels of the 5 OXPHOS complexes (complexes I, II, III, IV and V) by WB.

### 2.7 Lactate dehydrogenase (LDH) release assay

The effects of PP on the THP-1 cytotoxicity and the 50% cytotoxicity concentrations (CC50, the concentration of PP that reduces cell viability by 50%) were determined using LDH release assay. Briefly, THP-1-derived macrophages were plated in 96-well plates, and infected with or without H37Ra for 4 h, then treated with different concentrations of PP as indicated for 24 h. The release of LDH was determined using a CytoTox 96 NonRadioactive Cytotoxicity Assay (Promega, USA). Cell viability curves for calculating CC50 are fitted using GraphPad (GraphPad Software, LLC., USA). The experiments were performed in triplicate.

### 2.8 Intracellular colony forming unit determination

To determine whether PP inhibits intracellular *M.tb* growth in macrophages, THP-1 cells were seeded in 12-well plates at a density of 5 × 10^5^ cells per well and treated with 100 ng/ml PMA for 24 h. Cells were infected with *M.tb* strains (H37Ra or H37Rv) at an MOI of 10 for 4 h, then cells were washed three times with PBS, and then fresh medium containing PP or DMSO was added. Plates were incubated at 37°C in 5% CO_2_ for 24 h. Cells were lysed with 0.1% Triton X-100. Viable bacteria were grown in serial dilutions on 7H10 agar plates. CFUs were counted after four weeks of incubation at 37°C.

### 2.9 Statistical analysis

Data are presented as the mean ± SD. Student’s t test was used for all statistical analyses with GraphPad Prism 8.0 software. Differences between groups were considered statistically significant when P values less than 0.05.

## 3 Results

### 3.1 PP suppresses NLRP3 expression and NLRP3–ASC–Casp-1 inflammasome activation in *M.tb*-infected macrophage

In our previous research, we have confirmed that PP could effectively inhibit inflammatory pathological changes in *M.tb* H37Rv-infected mice (21). Thus, we measured five capital human inflammasome molecules (NLRP1, NLRP3, NLRC4, AIM2 and Pyrin) mRNA expression in H37Ra-infected human THP-1 macrophages at different time points. We found that H37Ra infection could only significantly increase NLRP3 mRNA expression in THP-1 cells (**Supplementary Figure 1A**). Then we measured the protein expression of NLRP3 in THP-1 cells after infecting with H37Ra at different MOI. The results showed that a dose-dependent elevation in NLRP3 expression as the infection dose increased (**Supplementary Figure 1B**). We chose an MOI of 10 as the infectious dose for subsequent experiments.

**Figure 1:**
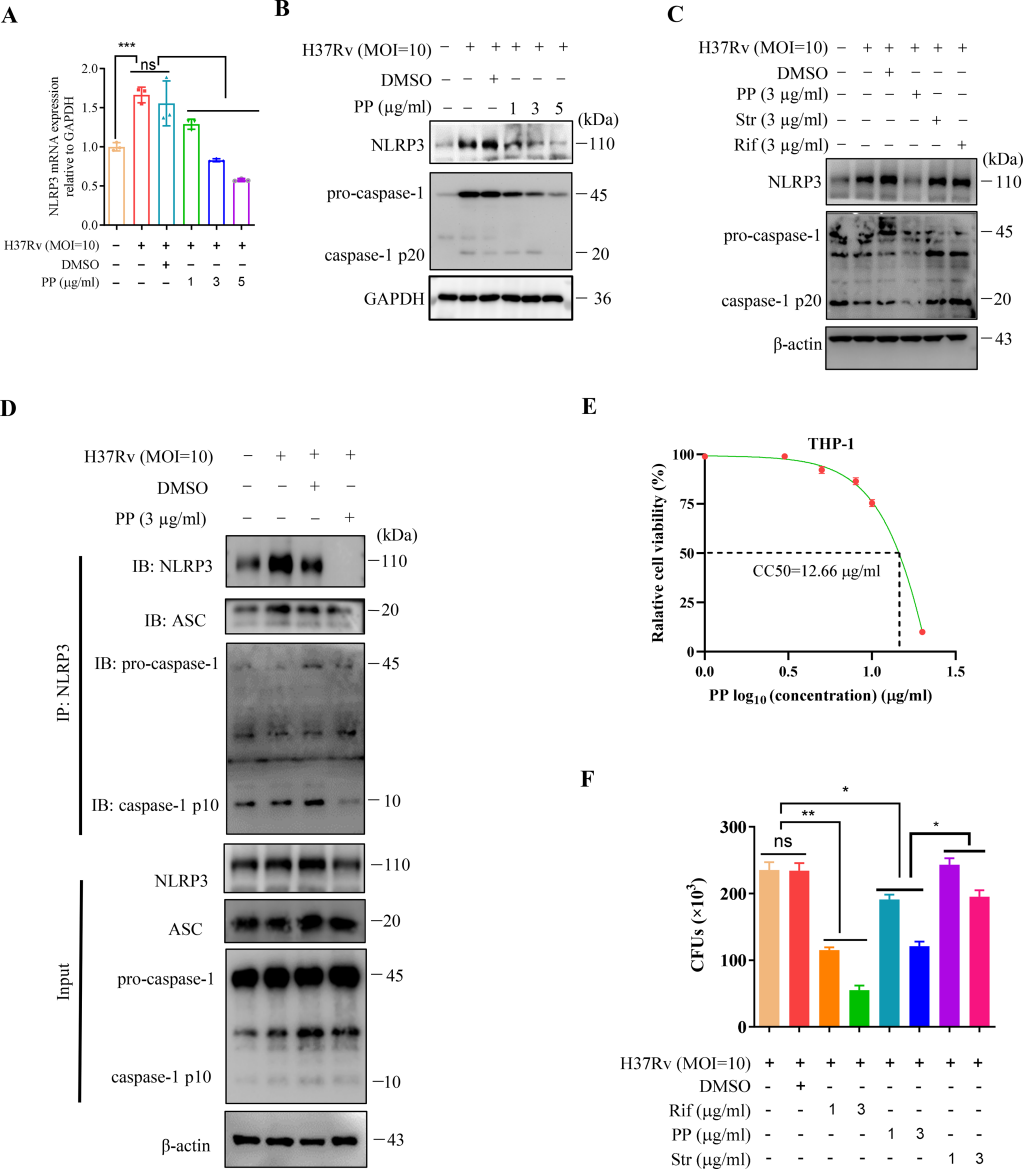
PP suppresses NLRP3–ASC–Casp-1 inflammasome activation in *M.tb* H37Rv-infected THP-1 cells. **(A)** RT-qPCR assay of the mRNA expression of NLRP3 in THP-1 cells. THP-1 cells were infected with H37Rv (MOI=10) for 4 h and then treated with different concentrations of PP for another 24 h. **(B)** WB assay of the NLRP3 and pro-caspase-1 protein expression in THP-1 cells. THP-1 cells were infected with H37Rv (MOI=10) for 4 h and then treated with different concentration of PP for another 24 h. **(C)** WB assay of the NLRP3 and pro-caspase-1 protein expression in THP-1 cells. THP-1 cells were infected with H37Rv (MOI=10) for 4 h and then treated with PP, Str and Rif for another 24 h. Str, streptomycin; Rif, Rifampicin. **(D)** THP-1 cells infected with H37Rv (MOI=10) for 4 h and then treated with PP for another 24 h were subjected to IP with anti-NLRP3 antibody and IB with antibodies against NLRP3, ASC, pro-caspase-1 and caspase-1 p10. **(E)** Relative cell viabilities of THP-1 after treatment with different concentrations of PP. **(F)** CFUs of M.tb H37Rv in THP-1 cells after PP, Str or Rif treatment. THP-1 cells were infected with H37Rv (MOI=10) for 4 h and then treated with PP, Str or RIF for 24 h. Str, streptomycin; Rif, Rifampicin. Data represent mean ± SD for three independent experiments. One-way ANOVA followed by Tukey’s multiple comparison test was used to assess the statistical difference for **(A, F)**. *P < 0.05; **P < 0.01.

Next, we detected the effect of PP on NLRP3 mRNA and protein expression in THP-1 cells infected with *M.tb* H37Rv. THP-1 cells were infected with *M.tb* H37Rv (MOI = 10) for 4 h and subsequently treated with PP for 24 h. As we expected, PP inhibits H37Rv-induced elevated expression of NLRP3 mRNA (**Figure 1A**) and protein (**Figure 1B**) expression in THP-1 cells in a dose-dependent manner. Otherwise, we also found that PP could inhibit H37Rv -induced elevated expression of NLRP3 in THP-1 cells, rifampicin and streptomycin could not (**Figure 1C**). Above results strongly demonstrate that PP could inhibit *M.tb* H37Rv/H37Ra-induced elevated NLRP3 expression in THP-1 cells.

Assembly of the NLRP3 inflammasome leads to activation of caspase-1 (22). We have found that PP inhibits *M.tb*-induced elevated expression of NLRP3, and thus we seek to determine if PP affects the assembly of the NLRP3 inflammasome and activation of caspase-1. Then immunoprecipitation and WB assay was used to detected the NLRP3 inflammasome assembly (NLRP3-ASC, Casp-1) and we found that PP significantly inhibited assembly of NLRP3 inflammasome (NLRP3-ASC-Casp-1) in H37Rv-infected THP-1 cells (**Figure 1D**).

In addition, we found that PP also suppresses *M.tb* H37Ra-induced elevated expression of NLRP3 mRNA (**Supplementary Figure 1C**) and protein (**Supplementary Figure 1D**) expression, and *M.tb* H37Ra-induced activation of caspase-1 in THP-1 cells in a PP drug dose-dependent manner (**Supplementary Figure 1D**), and PP significantly inhibited assembly of NLRP3 inflammasome (NLRP3-ASC-Casp-1) in *M.tb* H37Ra-infected THP-1 cells (**Supplementary Figure 1E**).

These results suggest that PP suppresses NLRP3 expression and NLRP3–ASC–Casp-1 inflammasome assembly in *M.tb*-infected macrophage.

### 3.2 PP inhibits intracellular *M.tb* survival in THP-1-derived macrophages

We have previously determined that the CC50 value of PP (CC50, the concentration that reduces the number of viable cells by 50%) on mouse bone marrow derived macrophages (BMDMs) was determined to be 2.4 μg/mL (21). Here, we further ascertained the cytotoxic effects of PP on human THP-1-derived macrophages by assessing the CC50, and found that the CC50 value of PP on THP-1-derived macrophages is 12.66 µg/mL (95% confidence interval, 9.249 to 17.33 µg/ml) (**Figure 1E**). We selected PP concentrations within 1~5 µg/ml range, below the value of PP CC50 (12.66 µg/ml), for subsequent THP-1 cells experiments. Within this concentration range (1~5 µg/ml), PP causes little or no cytotoxicity.

In our previous research, we have demonstrated that PP could directly kill the *M.tb* H37Rv *in vitro* (21). However, whether PP could inhibit intracellular *M.tb* survival remains unknown yet. In order to confirm that PP could inhibit intracellular *M.tb* survival, we performed intracellular CFUs counting assay for *M.tb* H37Rv infected-THP-1 cells in the presence or absence of PP, and found that PP inhibited the intracellular *M.tb* H37Rv/H37Ra survival in THP-1 cells in a PP drug dose dependent manner (**Figure 1F**, **Supplementary Figure 1F**). These results clearly suggest that PP inhibits the intracellular *M.tb* survival in THP-1 macrophages with dose dependence.

### 3.3 PP suppresses *M.tb-*, LPS- and *S. typhimurium*-induced GSDMD/IL-1β/IL-18 in THP-1

**Figure 2:**
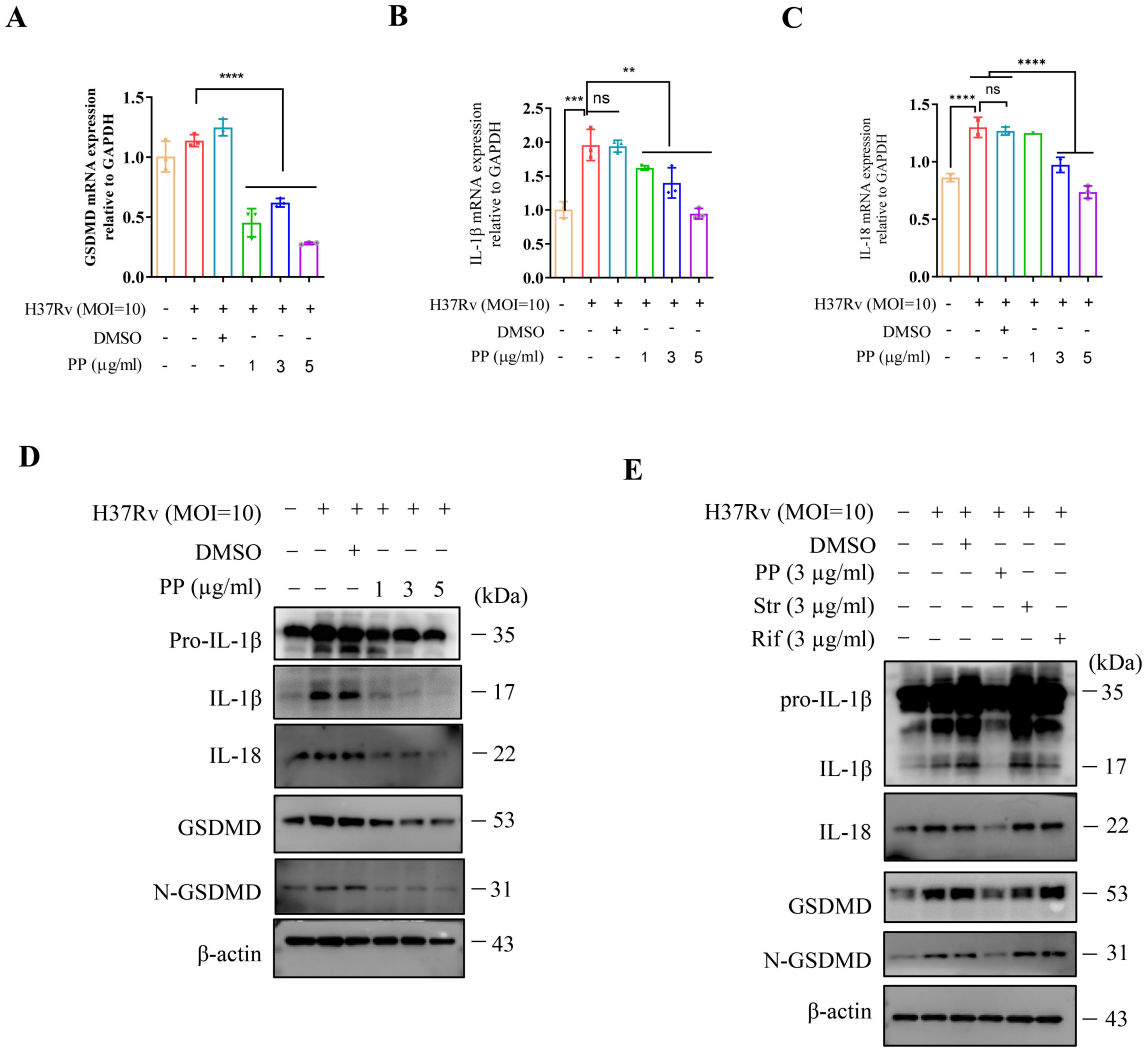
PP suppresses *M.tb* H37Rv-induced GSDMD cleavage and IL-1β production in THP-1 cells. **(A-C)** RT-qPCR assay of GSDMD **(A)**, IL-1β **(B)** and IL-18 **(C)** mRNA expression in THP-1 cells. THP-1 cells were infected with H37Rv (MOI=10) for 4 h and then treated with different concentrations of PP for another 24 h. **(D)** WB assay of pro-IL-1β, IL-1β, IL-18, GSDMD and N-GSDMD protein expression in THP-1 cells. THP-1 cells were infected with H37Rv (MOI=10) for 4 h and then treated with different concentrations of PP for another 24 h. **(E)** WB assay of pro-IL-1β, IL-1β, IL-18, GSDMD and N-GSDMD protein expression in THP-1 cells. THP-1 cells were infected with H37Rv (MOI=10) for 4 h and then treated with PP, Str or Rif for another 24 h. Str, streptomycin; Rif, Rifampicin. Data represent mean ± SD for three independent experiments. One-way ANOVA followed by Tukey’s multiple comparison test was used to assess the statistical difference for **(A<C)**. **P < 0.01; ****P < 0.0001.

As demonstrated by our studies and those of other research groups, *M.tb* infection could induce cell inflammatory pyroptosis, and N-GSDMD cleavage and IL-1β secretion were significantly evaluated (23–25). The marker for pyroptosis occurrence is the GSDMD expression and cleavage (N-GSDMD). The effect of PP on the expression of GSDMD/IL-1β/IL-18 in *M.tb*-infected THP-1 was measured. As shown in **Figures 2A-C**, PP significantly inhibited H37Rv-induced elevated expression of GSDMD, pro-IL-1β and IL-18 mRNA in THP-1 cells in a dose-dependent manner. Meanwhile, we also found that PP inhibited the maturation of GSDMD, IL-1β and IL-18 (**Figure 2D**) in *M.tb* H37Rv-infected THP-1 cells. And only PP could inhibit the cleavage of GSDMD (as shown by the decreased N-GSDMD fragment), and the elevation of IL-1β and IL-18 expression in *M.tb* H37Rv-infected THP-1 cells. However, rifampicin and streptomycin exhibited no these effects (**Figure 2E**).

Then we measured the protein expression of N-GSDMD and IL-1β in THP-1 cells after infecting with *M.tb* H37Ra. we also found that PP significantly inhibited H37Ra-induced elevated expression of GSDMD, pro-IL-1β and IL-18 mRNA and protein expression in THP-1 cells in a PP dose-dependent manner (**Supplementary Figures 2A-E**). Subsequently, PP significantly inhibited *M.tb* H37Ra*-*induced pyroptosis via LDH assay (**Supplementary Figure 2F**).

We have proved in the above section that PP could inhibit *M.tb*-induced elevated expression of NLRP3, but it remains to be determined whether PP could also inhibit the potential elevated expression of NLRP3 in THP-1 cells induced by LPS or infected with *S. typhimurium* C5. Then we detected the expression of NLRP3 in THP-1 cells. The results showed that LPS could dose-dependently promote the expression of NLRP3/IL-1β in THP-1 cells (**Supplementary Figure 3A**). As expected, PP could also inhibit LPS/*S. typhimurium* C5-induced elevated expression of NLRP3 in THP-1 cells (**Supplementary Figures 3B, C**). These results further confirmed that PP could inhibit the elevated NLRP3 expression in THP-1 cells induced by both LPS and *S. typhimurium* C5. We also found that PP could inhibit LPS- and *S. typhimurium* C5-induced GSDMD/IL-1β in THP-1 cells (**Supplementary Figures 3B, C**).

Above results indicate that PP inhibits the assembly of the NLRP3 inflammasome, and thus decreases the activation of caspase-1, which inhibits the maturation and release of GSDMD/IL-1β/IL-18, and finally inhibits *M.tb*, LPS or *S. typhimurium-*induced inflammatory pyroptosis.

### 3.4 PP suppresses *M.tb*-induced NLRP3 inflammasome activation via β-catenin–NF-κB signal pathway

**Figure 3:**
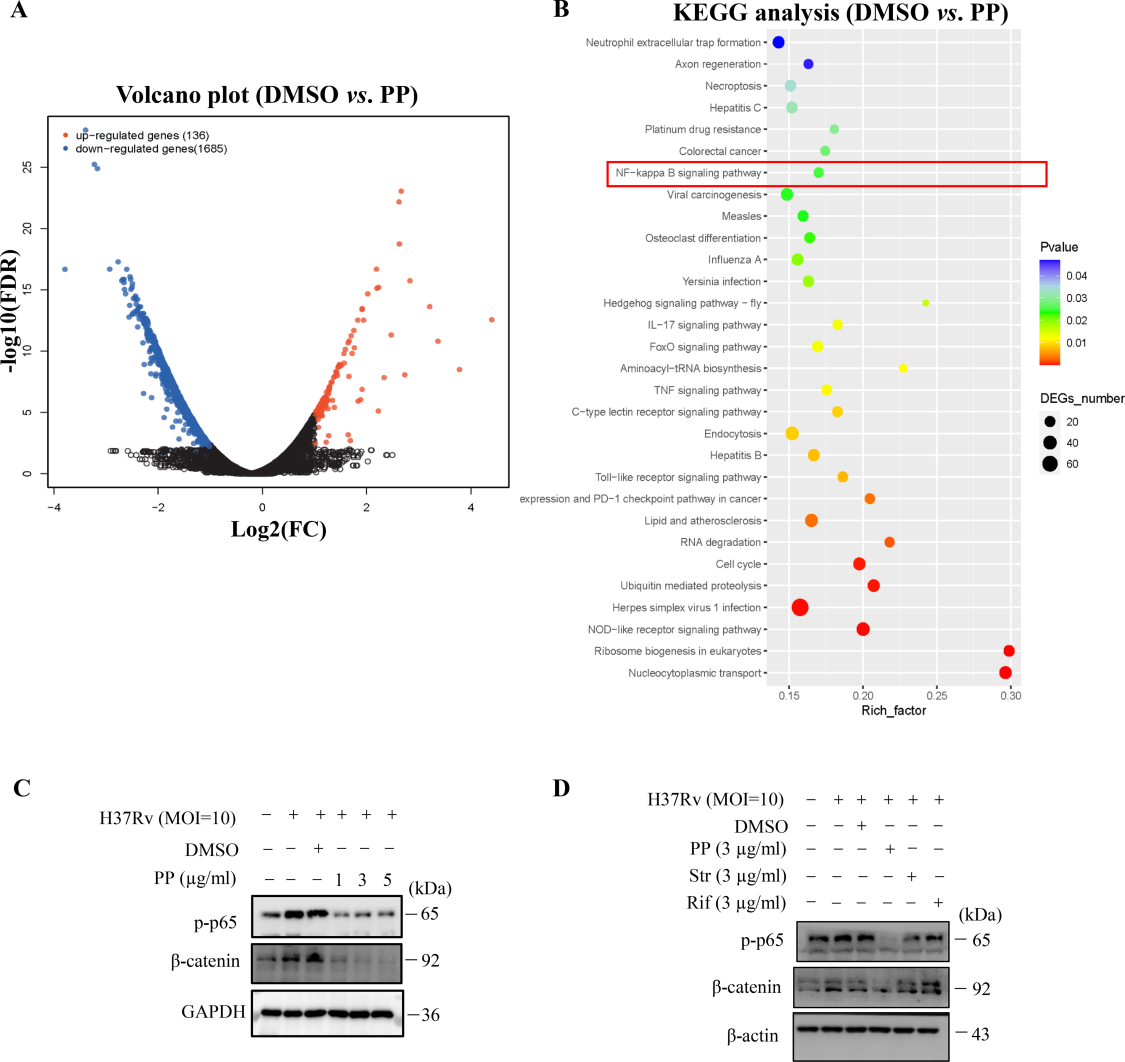
PP suppresses *M.tb* H37Rv-induced NLRP3 activation via repressing the β-catenin–NF-κB signal pathway. **(A)** Volcano plot of RNA-seq results. **(B)** KEGG enrichment of differentially expressed genes identified in transcriptome sequencing of H37Rv-infected THP-1 cells that were treated with PP. **(C)** WB assay of p-p65 and β-catenin protein expression in THP-1 cells. THP-1 cells were infected with H37Rv (MOI=10) for 4 h and then treated with different concentrations of PP for another 24 h. **(D)** WB assay of p-p65 and β-catenin protein expression in THP-1 cells. THP-1 cells were infected with H37Rv (MOI=10) for 4 h and then treated with PP, Str or Rif for another 24 h. PP, pyrvinium pamoate; Str, streptomycin; Rif, Rifampicin.

As is well-documented, NF-κB activation is required for the expression of NLRP3, GSDMD and IL-1β (26–28). The KEGG enrichment analysis of transcriptome sequencing results showed that PP affected the NF-κB signaling pathway in H37Rv-infected THP-1 cells (**Figures 3A, B**). Therefore, we investigated whether PP impairs *M.tb*-induced NF-κB activation, and we found that *M.tb* H37Rv infection could promote the activation of NF-κB (**Figure 3C**). H37Rv infection promoted the phosphorylation of NF-κB in THP-1 cells, but the phosphorylation level of NF-κB in THP-1 cells was significantly decreased after PP treatment (**Figure 3C**). Furthermore, only PP could inhibit H37Rv-induced elevated phosphorylation level of NF-κB in THP-1 cells, and rifampicin and streptomycin could not (**Figure 3D**).

Studies have shown that microbiota could induce inflammatory responses by activating the NF-κB pathway through β-catenin (29). Thus, we analyzed the effects of PP on β-catenin. Our results showed that *M.tb* H37Rv infection could promote β-catenin expression (**Figure 3C**), but significantly decreased when treated with PP (**Figure 3C**). Furthermore, only PP could inhibit H37Rv-induced elevated expression of β-catenin in THP-1 cells, and rifampicin and streptomycin could not (**Figure 3D**).

We also found that *M.tb* H37Ra infection could promote the activation of NF-κB (**Supplementary Figure 4A, B**), and the phosphorylation of NF-κB in THP-1 cells, but the phosphorylation level of NF-κB in THP-1 cells was significantly decreased when treated with PP (**Supplementary Figure 4B**). Our results also showed that *M.tb* H37Ra infection could promote β-catenin expression (**Supplementary Figure 4A, B**), but significantly decreased when treated with PP (**Supplementary Figure 4B**).

We also found that PP inhibits LPS- and *S. typhimurium* C5-induced elevated expression of NF-κB in THP-1 cells (**Supplementary Figures 4C, D**). At the same time, we also found that PP inhibits LPS- and *S. typhimurium C5*-induced elevated expression of β-catenin in THP-1 cells (**Supplementary Figures 4C, D**).

MSAB (methyl 3-{[(4-methylphenyl)sulfonyl]amino}benzoate), a β-catenin inhibitor, could inhibits H37Ra-induced elevated expression of p-p65 and NLRP3 (**Supplementary Figure 4E**), which means that β-catenin is upstream of NF-κB. Moreover, pyrrolidine dithiocarbamate (PDTC), a NF-κB inhibitor, exhibited no inhibition on β-catenin expression (**Supplementary Figure 4F**). All these results indicate that PP inhibits NF-κB activation through suppressing β-catenin.

### 3.5 PP suppresses *M.tb*-induced NLRP3 inflammasome activation via NRF2–mitochondrial OXPHOS pathway as well

**Figure 4:**
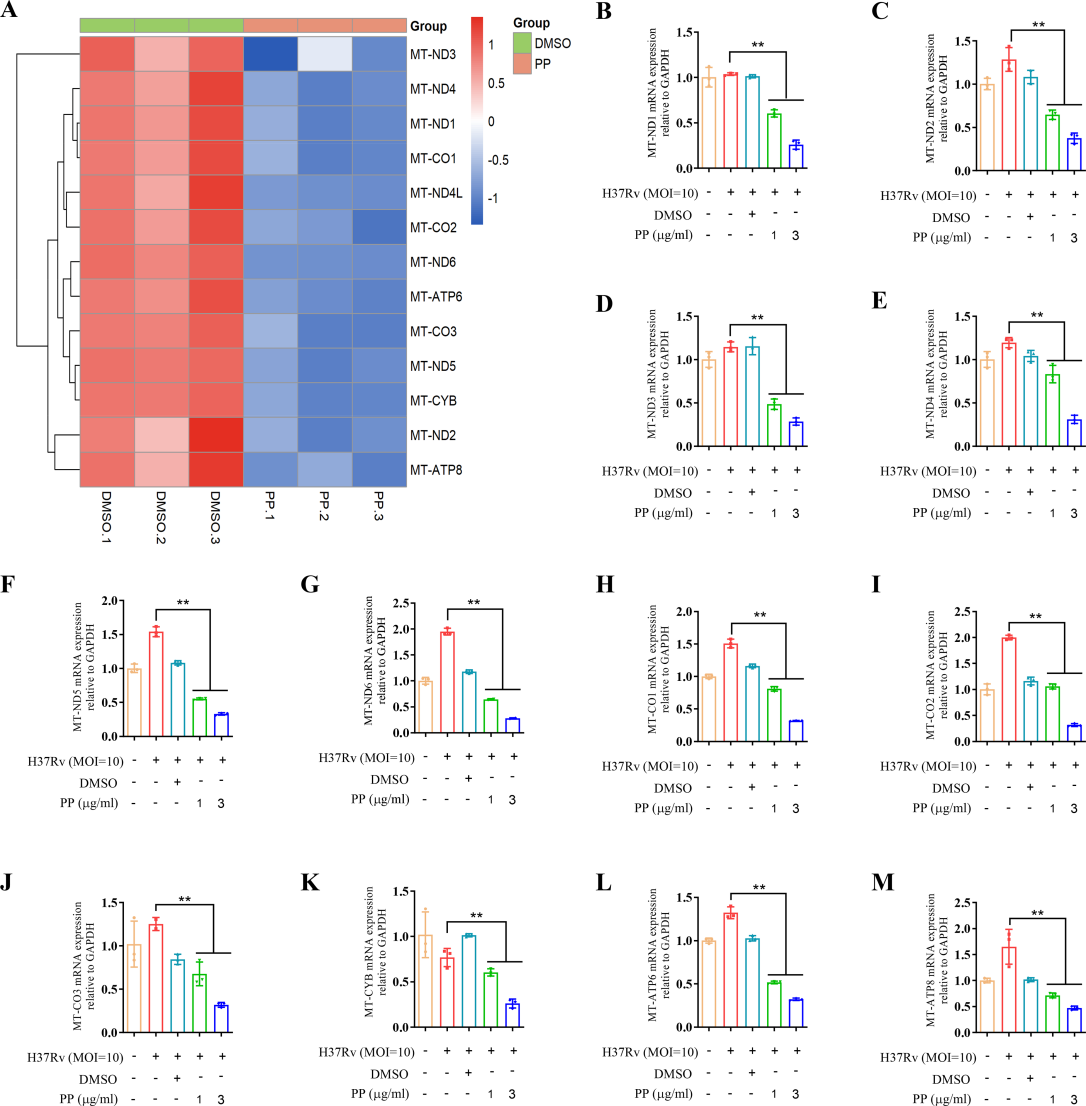
PP suppresses mitochondrial OXPHOS-related mRNA expression in H37Rv-infected THP-1 cells. **(A)** Heatmap of differentially expressed mitochondrial OXPHOS-related genes identified in transcriptome sequencing of THP-1 cells. THP-1 cells were infected with H37Rv (MOI=10) for 4 h and then treated with PP at 3 µg/ml for another 24 h. **(B-M)** RT-qPCR verification of mitochondrial OXPHOS-related mRNA expression. Data represent mean ± SD for three independent experiments. One-way ANOVA followed by Tukey’s multiple comparison test was used to assess the statistical difference for B-M. **P < 0.01.

The transcriptome sequencing results showed that PP significantly inhibited the mRNA expression of mitochondrial oxidative phosphorylation (OXPHOS) -related genes in THP-1 cells after infecting with H37Rv (**Figure 4A**). And qRT-PCR confirmed that PP could inhibit the mRNA expression of mitochondrial OXPHOS-related genes (mitochondrially encoded NADH dehydrogenase (MT-ND)1/2/3/4/5/6, MT cytochrome C oxidase (MT-CO)1/2/3, MT cytochrome B (MT-CYB), MT ATP synthase membrane subunit (MT-ATP)6/8) (**Figures 4B-M**).

**Figure 5:**
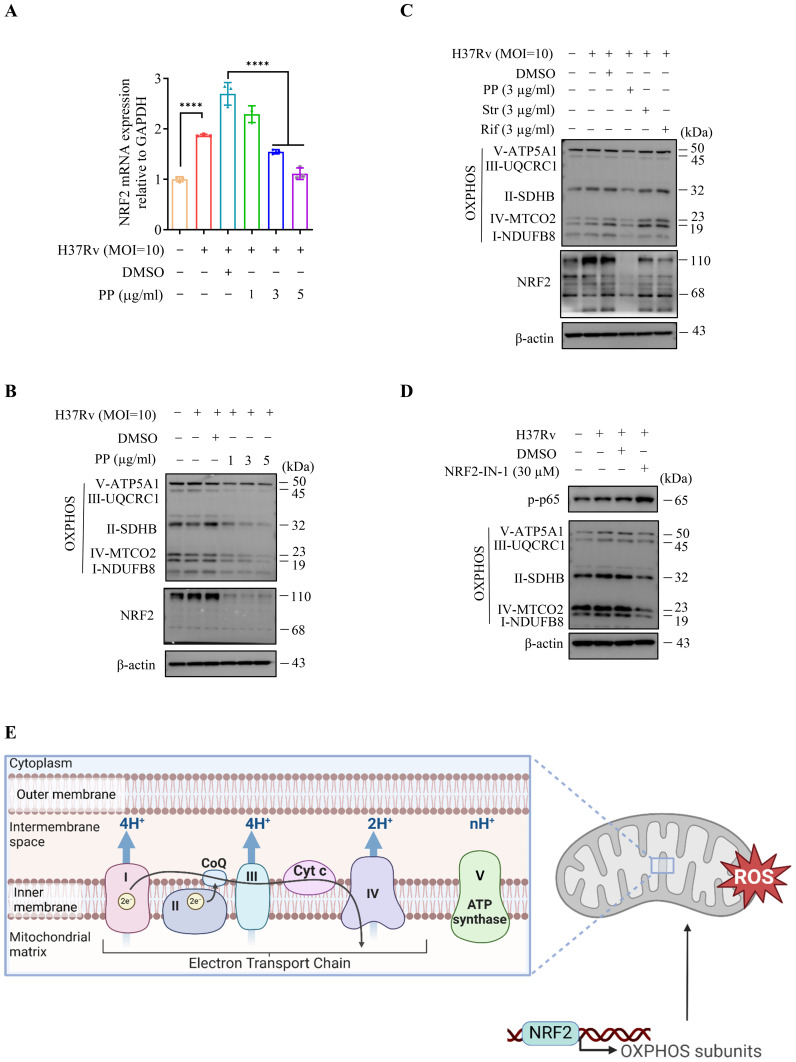
PP inhibits NRF2–mitochondrial OXPHOS pathway during H37Rv infection in macrophages. **(A)** RT-qPCR assay of NRF2 mRNA expression in THP-1 cells that infected with H37Rv(MOI=10) for 4 h and then treated with different concentrations of PP for another 24 h. **(B)** WB assay of NRF2 and mitochondrial OXPHOS-related protein expression in THP-1 cells. THP-1 cells were infected with H37Rv (MOI=10) for 4 h and then treated with different concentrations of PP for another 24 h. **(C)** WB assay of NRF2 and mitochondrial OXPHOS-related protein expression in THP-1 cells. THP-1 cells were infected with H37Rv (MOI=10) for 4 h and then treated with PP, Str or Rif for another 24 h. Str, streptomycin; Rif, Rifampicin. **(D)** WB assay of p-p65 and OXPHOS-related protein expression in THP-1 cells. THP-1 cells were infected with H37Rv (MOI=10) for 4 h and then treated with NRF2-IN-1 (NRF2 inhibitor) at 30 µM for another 24 h. **(E)** Schematic of NRF2 regulating OXPHOS-related gene expression and thus upregulating ROS. Data represent mean ± SD for three independent experiments. One-way ANOVA followed by Tukey’s multiple comparison test was used to assess the statistical difference for A. ****P < 0.0001.

As previous reports showed that NF-κB could promote NRF2 transcription (30), we assessed the effects of PP on NRF2 and mitochondrial OXPHOS-related protein expression in *M.tb*-infected THP-1 cells. The results showed that PP inhibited NRF2 mRNA expression (**Figure 5A**), NRF2 and mitochondrion OXPHOS (I-NDUFB8, II-SDHB, III-UQCRC1, IV-MTCO2 and V-ATP5A1) protein expression in H37Rv-infected THP-1 cells (**Figure 5B**). And only PP could inhibit NRF2 and mitochondrion OXPHOS in *M.tb* H37Rv-infected THP-1 cells, and rifampicin and streptomycin could not (**Figure 5C**). At the same time, we also assessed whether NRF2-IN-1 (NRF2 inhibitor) could suppress the effect of M.tb on OXPHOS. As we expected, the results showed that NRF2-IN-1 could inhibit *M.tb* H37Rv-induced (**Figure 5D**) elevated expression of OXPHOS, which means that NRF2 is upstream of OXPHOS.

In addition, we also measured mitochondrion OXPHOS in THP-1 cells after infecting with H37Ra at different MOI, and the results showed that mitochondrion OXPHOS elevated along with the increase of infection dose (**Supplementary Figure 4A**). And PP inhibited NRF2 and mitochondrion OXPHOS protein expression in H37Ra -infected THP-1 cells (**Supplementary Figure 4B**).

We also found that PP inhibited LPS- and *S. typhimurium* C5-induced NRF2 and mitochondrion OXPHOS in THP-1 cells (**Supplementary Figures 4C, D**).

We further assessed whether PDTC (NF-κB inhibitor) and MSAB (β-catenin inhibitor) could suppress the effects of H37Ra on NRF2 expression. Our results showed that MSAB (β-catenin inhibitor) could inhibit H37Ra-induced elevated expression of NRF2 (**Supplementary Figure 4E**), which means that β-catenin is upstream of NRF2. Moreover, PDTC (NF-κB inhibitor) exhibited inhibition on H37Ra-induced elevated NRF2 expression (**Supplementary Figure 4F**). And NRF2-IN-1 could inhibit *M.tb* H37Ra-induced expression of OXPHOS (**Supplementary Figure 4G**). These results confirm PP inhibits *M.tb*-induced β-catenin–NF-κB–NRF2 signaling pathway.

Oxidative phosphorylation (OXPHOS) of mitochondria is an important source of reactive oxygen species (ROS) (30), and ROS production is the second signal for NLRP3 activation (14, 15). Our above results indicate that PP inhibits *M.tb*, LPS and *S. typhimurium* C5-induced mitochondrion OXPHOS in THP-1 cells, which subsequently suppresses ROS production (**Figure 5E**), and finally inhibits the activation of NLRP3.

Taken together, these results indicate that PP inhibits NRF2–mitochondrial OXPHOS pathway during mycobacterial infection in macrophages.

### 3.6 PP exhibits suppressive effects through targeting CK1α

**Figure 6:**
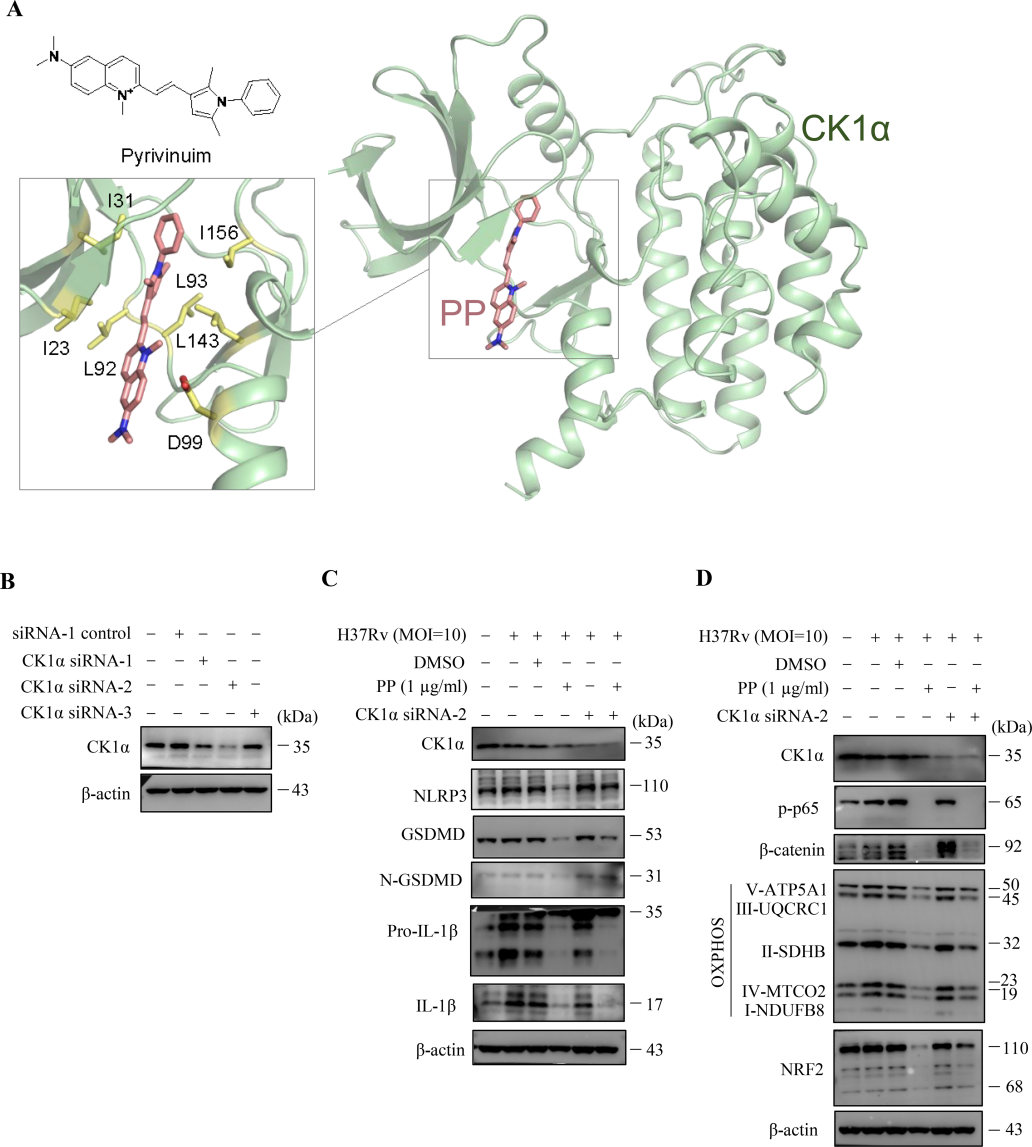
CK1α siRNA reverses the inhibitory effects of PP on *M.tb* H37Rv-induced inflammatory responses in macrophages. **(A)** Docking model of CK1α and pyrvinium. Protein CK1α is shown as cartoon, the ligand pyrvinium and the key ligand-binding residues are shown as sticks. **(B)** WB assay of CK1α protein expression in THP-1 cells that were transfected with each of the three CK1α siRNAs, respectively. **(C)** WB assay of CK1α, NLRP3, GSDMD, N-GSDMD, pro-IL-1β and IL-1β protein expression in H37Rv-infected THP-1 cells that were treated with/without PP and/or CK1α siRNA-2. Cells were transiently transfected for 24 h with CK1α siRNA-2 and then replaced with fresh culture medium and cultured for another 12 h. Then H37Rv was respectively used to infect THP-1 cell for 4 h, the culture was abandoned and cells were washed with PBS for 3 times, and then PP was added and cultured for another 24 h. **(D)** WB assay of CK1α, p-p65, β-catenin, NRF2 and mitochondrial OXPHOS-related protein expression in H37Rv-infected THP-1 cells that were treated with/without PP and/or CK1α siRNA-2.

Previous reports showed that PP could reduce β-catenin levels by selectively potentiating casein kinase 1α (CK1α) kinase activity to degrade β-catenin (31, 32). We conducted molecular docking of small compound PP and protein kinase CK1α, and the results showed that PP potentially interacts with I23, I31, L92, L93, D99, L143 and L156 residues which are located in the protein kinase domain of CK1α (**Figure 6A**). The isoleucine (I23, I31) and leucine (L92, L93, L143, L156) residues, which are involved in hydrophobic interactions, provide a stable binding environment for PP. Aspartic acid (D99) introduces an element of electrostatic interaction into the binding site. This negatively charged residue can form hydrogen bonds or salt bridges with positively charged groups on PP. The isoleucine (I23, I31) resides in the ATP-binding site on CK1α, and the interacting residues (I23, I31, L92, L93, D99, L143 and L156) are spatially proximal to the active site (D136) of CK1α, thus binding of PP to CK1α might induce a conformational change, exposing the CK1α active site and allosterically activating CK1α. This suggests that PP can potentially bind to key regions of the kinase domain, which may influence the enzymatic activity or regulatory functions of protein CK1α.

In order to further verify that the target of PP is CK1α, three CK1α-specific siRNAs were synthesized (**Supplementary Table 2**) and the results showed that only CK1α siRNA2 could significantly inhibit the expression of CK1α (**Figure 6B**). We utilized CK1α siRNA2 for silencing CK1α, and found that CK1α knockdown did reverse the inhibitory effect of PP on NLRP3–GSDMD–IL-1β inflammatory pyroptosis in H37Rv-infected THP-1 cells (**Figure 6C**). We also found that CK1α knockdown could reverse the inhibitory effect of PP on β-catenin, NRF2 and OXPHOS expression in H37Rv-infected THP-1 cells (**Figure 6D**), albeit there was no significant change in p-p65 levels, possibly due to the involvement of other pathways in inhibiting p65 levels. These results further suggest that PP exhibits suppressive effects through targeting CK1α.

Further, D4476, a CK1α specific inhibitor, was used to verify whether the inhibition effect of PP on NLRP3**–**GSDMD**–**IL-1β inflammatory pyroptosis could be reversed. As we expected, D4476 did reverse the inhibition effect of PP on NLRP3**–**GSDMD**–**IL-1β inflammatory pyroptosis in H37Ra infected THP-1 cells (**Supplementary Figures 5A, B**). We also found that D4476 could reverse the inhibition effect of PP on β-catenin and p-p65 expression in H37Ra infected THP-1 cells (**Supplementary Figure 5C**). Further we found that D4476 could reverse the inhibition effect of PP on NRF2 and OXPHOS expression in H37Ra-infected THP-1 cells (**Supplementary Figure 5C**). These data suggest that PP exhibits suppressive effects through targeting CK1α.

These above results suggest that CK1α siRNA or specific inhibitor D4476 could reverse these inhibitory effects of PP on mycobacteria-induced inflammatory responses in macrophages through alleviating PP suppression of CK1α–β-catenin/NF-κB signal pathway and CK1α–NRF2–mitochondrial OXPHOS pathway.

## 4 Discussion

**Figure 7:**
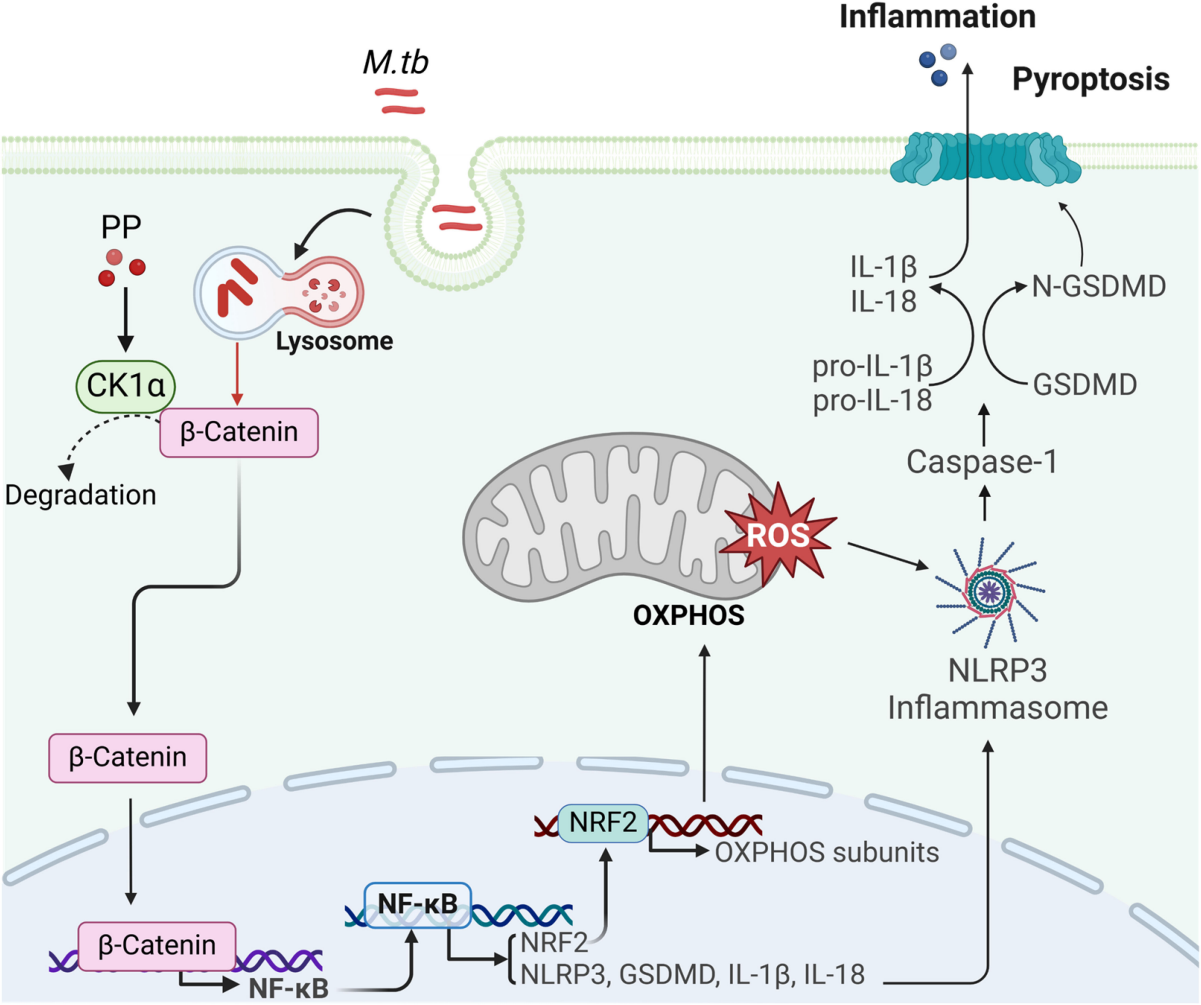
The mechanism of PP suppressing NLRP3 inflammasome by targeting CK1α–β-catenin–NF-κB–NRF2–mitochondrial OXPHOS pathways during bacterial infection. M.tb could induce NLRP3–ASC–Casp-1 inflammasome activation and GSDMD–IL-1β inflammatory pyroptosis through suppressing casein kinase 1A1 (CK1α)–β-catenin/NF-κB signaling pathway and CK1α–NRF2–mitochondrial oxidative phosphorylation (OXPHOS) pathway. PP could suppress this effect by targeting CK1α.

TB is the world’s second leading cause of death from a single infectious agent in 2024, which seriously threatens public health (1). Traditional treatment of TB with antibiotics all aims to directly kill *M.tb*, but long-term use of antibiotics is responsible for drug resistance. The WHO reported that in 2023, the proportion of multidrug-resistant/rifampicin resistant patients among new patients is 3.7% (1), which slightly higher than 3.3% in 2022. Drug resistance, latent infection, and co-infection with human immunodeficiency virus (HIV) are the main challenges for the treatment of TB. Thus, a new adjuvant therapy strategy has emerged and developed, namely HDT. HDT aims to either directly enhance host immunity for controlling infected *M.tb* or to minimize tissue damage caused by excessive inflammation (9). As a cyanine, PP has a series of biological activities, such as anthelminthic (33), anti-tumor (18), directly kill *M.tb* (21), anti-fungus (34, 35), anti-cryptosporidium (36), and anti-inflammatory (19–21, 37). In the present research, the anti-inflammatory pyroptosis activity of PP was evaluated in *M.tb*-infected THP-1 macrophage. The results revealed that PP inhibits *M.tb*-induced NLRP3-GSDMD-IL-1β inflammatory pyroptosis via suppressing β-catenin/NF-κB pathway and ROS production. Moreover, this inhibitory activity of PP was primarily mediated by binding with CK1α and promoting its activity to degrade β-catenin (**Figure 7**).

PP exerts the therapeutic function through multiple mechanisms: (a) interfering with the respiratory system of helminths, inhibiting the uptake of oxygen, increasing the anaerobic glycolysis of sugar and impeding the absorption of glucose, which causes the parasite to die, but does not kill the eggs (36). (b) Recently, PP has also been found to resist fungal infections by inducing apoptosis/necrosis, inhibiting drug efflux pumps, and modulating signaling pathways related with stress response and growth control (35). (c) Multiple researches have reported that PP exhibits anti-cancer effects. pyrvinium targets solid (e.g., prostate and breast) and blood (e.g., myeloma and leukemia) cancers via three main mechanisms: (i) Wnt–β-catenin pathway suppression; (ii) CK1α activation; and (iii) mitochondrial respiration inhibition (18). In the present study, our data reveal that PP could inhibits *M.tb*-induced NLRP3–GSDMD–IL-1β inflammatory pyroptosis via targeting CK1α–β-catenin–NF-κB and CK1α–NRF2–mitochondrial OXPHOS pathways. As our results showed that PP could also inhibit LPS (no bacteria)-induced NLRP3–ASC–Casp-1 inflammasome activation and GSDMD–IL-1β inflammatory pyroptosis, therefore the observed effects are not attributable to bacterial cytotoxicity or reduced bacterial viability leading to decreased expression of indicated proteins, pathways and pyroptosis.

Macrophages, the most important cell type among the host’s immune cells for killing *M.tb*, can produce large amounts of proinflammatory cytokines, including IL-1β. IL-1β is critical in the host defense against various kinds of bacteria and viruses (14), and the release and mature of IL-1β requires the activation of inflammasomes and GSDMD. The results of this study showed that PP inhibited the NLRP3–GSDMD–IL-1β inflammatory pyroptosis induced by *M.tb* infection. Furthermore, we found that LPS- and *S. typhimurium* C5-induced NLRP3–GSDMD–IL-1β inflammatory pyroptosis were also inhibited by PP.

NF-κB signaling and inflammasome-pyroptosis pathways act as critical part of innate immunity against pathogens including *M.tb* (25, 38–40). NF-κB activation contributes to the upregulation of NLRP3, GSDMD and pro-IL-1β (25, 38–40). The inflammatory response (5–8) or pyroptosis (41–44) is a two-edged sword in TB pathogenesis. Although inflammatory innate immunity or pyroptosis could control the infection, however the excessive inflammatory response (5–8) or excessive inflammatory pyroptosis (41–44) can lead to tissue damage, organ failure or even death. The results of this study showed that PP inhibited the *M.tb*-induced NLRP3 inflammasome (**Figures 1A-D**, **Supplementary Figures 1B-E**) and pyroptosis (**Figures 2D, E**, **Supplementary Figures 2B-F**), thereby suppressing potential excessive inflammatory response (21). At the same time, we also found that PP inhibits NF-κB pathway in LPS-treated or *S. typhimurium C5*-infected THP-1 cells. These results indicated that PP could suppress *M.tb*-, LPS- and *S. typhimurium* C5-induced NLRP3–GSDMD–pro-IL-1β inflammatory pyroptosis via NF-κB pathway. We also found that PP significantly inhibits intracellular *M.tb* survival, the detail mechanisms underlying its inhibitory effects on *M.tb* is currently being explored.

β-catenin regulates a variety of cellular processes, including inflammatory responses (45). Microbiota could activate NF-κB through the β-catenin to induce inflammatory response (29), and thus, we detected the effect of PP on the expression of β-catenin in *M.tb* infected THP-1 cells and found that PP also inhibits the expression of β-catenin in *M.tb* infected THP-1 with dose dependence. At the same time, we also found that PP inhibit the expression of β-catenin in LPS-treated and *S. typhimurium* C5-infected THP-1 cells. Otherwise, MSAB, a β-catenin inhibitor, could inhibit NF-κB in *M.tb*-infected THP-1 cells, which means that β-catenin is upstream of NF-κB. All of these results indicate that PP inhibits β-catenin–NF-κB signal pathway in LPS-treated, and *M.tb*- and *S. typhimurium* C5-infected THP-1 cells.

Furthermore, β-catenin is degraded by protein complexes, including AXIN, APC, GSK-1 and CK1α. Studies have shown that PP could binds CK1α and potentiates CK1α kinase activity, and finally inhibits β-catenin (31). Thus, we speculate that PP inhibits β-catenin–NF-κB signal pathway by binding CK1α and potentiating its activity, and then CK1 siRNA or D4476, a CK1α inhibitor, was used to verify our hypothesis. The results showed that CK1 siRNA or D4476 could reverse the inhibitory of PP on the elevation of NLRP3 expression in *M.tb*-infected THP-1 cells. These results indicate that PP inhibits β-catenin–NF-κB signal pathway by potentiating CK1α kinase activity, and finally suppresses *M.tb*-, LPS- and *S. typhimurium C5*-induced NLRP3–GSDMD–IL-1β inflammatory pyroptosis in THP-1 cells.

ROS production is the second signal for NLRP3 activation (14, 15), and mitochondrial OXPHOS is responsible for ROS production. As PP exhibits excitation at 500-550 nm and emission at 575-650 nm, which can interfere with the detection of ROS with DCFDA-based fluorescence ROS assay, we assessed the expression of multiple factors, such as those involved in mitochondrial OXPHOS, which determine the production of ROS. Our results shown that PP could inhibit *M.tb*-, LPS- and *S. typhimurium* C5-induced mitochondrial OXPHOS, and thus inhibit the expression of ROS production, and finally inhibit the NLRP3–GSDMD–IL-1β inflammatory pyroptosis in *M.tb*, LPS-treated and *S. typhimurium* C5-infected THP-1 cells. In our future studies, we will investigate whether PP could exhibit these effects *in vivo* with mouse infection models. Given that COVID-19 (46–48), other viral or bacterial infections (49–51), and conditions such as rheumatoid arthritis and gout (52, 53) can also lead to NLRP3 inflammasome activation and induce excessive immune responses, PP may also exert inhibitory effects on inflammation induced by these diseases.

Taken together, the findings of the present study demonstrated that PP inhibited the expression of NLRP3–GSDMD–IL-1β inflammatory pyroptosis in *M.tb*, LPS and *S. typhimurium C5* infected macrophages. The inhibitory activity of PP on NLRP3–GSDMD–IL-1β inflammatory pyroptosis is involved in the suppression of β-catenin-NF-κB signal pathway and ROS production (**Figure 7**). The findings of this study provide a more enhanced understanding between PP and *M.tb* infection, and reveal a novel mechanism of PP-mediated suppression of inflammatory pyroptosis. Our data may also provide a new HDT drug for *M.tb* infection and inflammatory pyroptosis.

## Data Availability

The datasets presented in this study can be found in online repositories. The names of the repository/repositories and accession number(s) can be found in the article/**Supplementary Material**.

## References

[1] World Health Organization. Global tuberculosis report 2024 . Available online at: https://www.who.int/teams/global-tuberculosis-programme/tb-reports/global-tuberculosis-report-2024 (Accessed November 20, 2024).

[2] O'Garra A Redford PS McNab FW Bloom CI Wilkinson RJ Berry MP. The immune response in tuberculosis. Annu Rev Immunol. (2013) 31:475–527. doi: 10.1146/annurev-immunol-032712-095939 23516984

[3] Ernst JD. The immunological life cycle of tuberculosis. Nat Rev Immunol. (2012) 12:581–91. doi: 10.1038/nri3259 22790178

[4] Tsenova L Singhal A. Effects of host-directed therapies on the pathology of tuberculosis. J Pathol. (2020) 250:636–46. doi: 10.1002/path.5407 32108337

[5] Monin L Khader SA. Chemokines in tuberculosis: the good, the bad and the ugly. Semin Immunol. (2014) 26:552–8. doi: 10.1016/j.smim.2014.09.004 PMC4314384 25444549

[6] Etna MP Giacomini E Severa M Coccia EM. Pro- and anti-inflammatory cytokines in tuberculosis: a two-edged sword in TB pathogenesis. Semin Immunol. (2014) 26:543–51. doi: 10.1016/j.smim.2014.09.011 25453229

[7] Mishra BB Rathinam VA Martens GW Martinot AJ Kornfeld H Fitzgerald KA. Nitric oxide controls the immunopathology of tuberculosis by inhibiting NLRP3 inflammasome-dependent processing of IL-1beta. Nat Immunol. (2013) 14:52–60. doi: 10.1038/ni.2474 23160153 PMC3721324

[8] Kirwan DE Chong DLW Friedland JS. Platelet activation and the immune response to tuberculosis. Front Immunol. (2021) 12:631696. doi: 10.3389/fimmu.2021.631696 34093524 PMC8170316

[9] Baindara P. Host-directed therapies to combat tuberculosis and associated non-communicable diseases. Microb Pathog. (2019) 130:156–68. doi: 10.1016/j.micpath.2019.03.003 30876870

[10] Martinon F Mayor A Tschopp J. The inflammasomes: guardians of the body. Annu Rev Immunol. (2009) 27:229–65. doi: 10.1146/annurev.immunol.021908.132715 19302040

[11] Paerewijck O Lamkanfi M. The human inflammasomes. Mol Aspects Med. (2022) 88:101100. doi: 10.1016/j.mam.2022.101100 35696786

[12] Song N Li T. Regulation of NLRP3 inflammasome by phosphorylation. Front Immunol. (2018) 9:2305. doi: 10.3389/fimmu.2018.02305 30349539 PMC6186804

[13] Broz P Dixit VM. Inflammasomes: mechanism of assembly, regulation and signalling. Nat Rev Immunol. (2016) 16:407–20. doi: 10.1038/nri.2016.58 27291964

[14] Yu X Lan P Hou X Han Q Lu N Li T. HBV inhibits LPS-induced NLRP3 inflammasome activation and IL-1beta production via suppressing the NF-kappaB pathway and ROS production. J Hepatol. (2017) 66:693–702. doi: 10.1016/j.jhep.2016.12.018 28027970

[15] Jo EK Kim JK Shin DM Sasakawa C. Molecular mechanisms regulating NLRP3 inflammasome activation. Cell Mol Immunol. (2016) 13:148–59. doi: 10.1038/cmi.2015.95 PMC4786634 26549800

[16] Ramachandran R Manan A Kim J Choi S. NLRP3 inflammasome: a key player in the pathogenesis of life-style disorders. Exp Mol Med. (2024) 56:1488–500. doi: 10.1038/s12276-024-01261-8 PMC11297159 38945951

[17] Green DR. Cell death: Revisiting the roads to ruin. Dev Cell. (2024) 59:2523–31. doi: 10.1016/j.devcel.2024.08.008 PMC11469552 39378838

[18] Momtazi-Borojeni AA Abdollahi E Ghasemi F Caraglia M Sahebkar A. The novel role of pyrvinium in cancer therapy. J Cell Physiol. (2018) 233:2871–81. doi: 10.1002/jcp.26006 28500633

[19] El-Derany MO El-Demerdash E. Pyrvinium pamoate attenuates non-alcoholic steatohepatitis: Insight on hedgehog/Gli and Wnt/beta-catenin signaling crosstalk. Biochem Pharmacol. (2020) 177:113942. doi: 10.1016/j.bcp.2020.113942 32240652

[20] Faheem SA El-Sayed NM Moustafa YM Saeed NM Hazem RM. Pyrvinium pamoate ameliorates cyclosporin A- induced hepatotoxicity via the modulation of Wnt/beta-catenin signaling and upregulation of PPAR-gamma. Int Immunopharmacol. (2022) 104:108538. doi: 10.1016/j.intimp.2022.108538 35074592

[21] Guan Q Zhan L Liu ZH Pan Q Chen XL Xiao Z. Identification of pyrvinium pamoate as an anti-tuberculosis agent *in vitro* and *in vivo* by SOSA approach amongst known drugs. Emerg Microbes Infect. (2020) 9:302–12. doi: 10.1080/22221751.2020.1720527 PMC7034053 32013776

[22] Xu J Nunez G. The NLRP3 inflammasome: activation and regulation. Trends Biochem Sci. (2023) 48:331–44. doi: 10.1016/j.tibs.2022.10.002 PMC10023278 36336552

[23] Beckwith KS Beckwith MS Ullmann S Saetra RS Kim H Marstad A. Plasma membrane damage causes NLRP3 activation and pyroptosis during Mycobacterium tuberculosis infection. Nat Commun. (2020) 11:2270. doi: 10.1038/s41467-020-16143-6 32385301 PMC7210277

[24] Yao Q Xie Y Xu D Qu Z Wu J Zhou Y. Lnc-EST12, which is negatively regulated by mycobacterial EST12, suppresses antimycobacterial innate immunity through its interaction with FUBP3. Cell Mol Immunol. (2022) 19:883–97. doi: 10.1038/s41423-022-00878-x PMC9149337 35637281

[25] Qu Z Zhou J Zhou Y Xie Y Jiang Y Wu J. Mycobacterial EST12 activates a RACK1-NLRP3-gasdermin D pyroptosis-IL-1β immune pathway. Sci Adv. (2020) 6(43):eaba4733. doi: 10.1126/sciadv.aba4733 33097533 PMC7608829

[26] Franchi L Munoz-Planillo R Nunez G. Sensing and reacting to microbes through the inflammasomes. Nat Immunol. (2012) 13:325–32. doi: 10.1038/ni.2231 PMC3449002 22430785

[27] Manna D Reghupaty SC Camarena MDC Mendoza RG Subler MA Koblinski JE. Melanoma differentiation associated gene-9/syndecan binding protein promotes hepatocellular carcinoma. Hepatology. (2022) 78(6):1727–41. doi: 10.1002/hep.32797 PMC11261751 36120720

[28] Liu Z Gan L Xu Y Luo D Ren Q Wu S. Melatonin alleviates inflammasome-induced pyroptosis through inhibiting NF-kappaB/GSDMD signal in mice adipose tissue. J Pineal Res. (2017) 63(1):e12414. doi: 10.1111/jpi.12414 28398673

[29] Yang L Li A Wang Y Zhang Y. Intratumoral microbiota: roles in cancer initiation, development and therapeutic efficacy. Signal Transduct Target Ther. (2023) 8:35. doi: 10.1038/s41392-022-01304-4 36646684 PMC9842669

[30] Wang Z Sun X Feng Y Wang Y Zhang L Wang Y. Dihydromyricetin reverses MRP2-induced multidrug resistance by preventing NF-κB-Nrf2 signaling in colorectal cancer cell. Phytomedicine. (2021) 82:153414. doi: 10.1016/j.phymed.2020.153414 33461143

[31] Thorne CA Hanson AJ Schneider J Tahinci E Orton D Cselenyi CS. Small-molecule inhibition of Wnt signaling through activation of casein kinase 1alpha. Nat Chem Biol. (2010) 6:829–36. doi: 10.1038/nchembio.453 PMC3681608 20890287

[32] Wang X Qin G Liang X Wang W Wang Z Liao D. Targeting the CK1alpha/CBX4 axis for metastasis in osteosarcoma. Nat Commun. (2020) 11:1141. doi: 10.1038/s41467-020-14870-4 32111827 PMC7048933

[33] Ishii I Harada Y Kasahara T. Reprofiling a classical anthelmintic, pyrvinium pamoate, as an anti-cancer drug targeting mitochondrial respiration. Front Oncol. (2012) 2:137. doi: 10.3389/fonc.2012.00137 23061049 PMC3462317

[34] Sun Y Gao L Zhang Y Yang J Zeng T. Synergistic effect of pyrvinium pamoate and azoles against aspergillus fumigatus *in vitro* and *in vivo* . Front Microbiol. (2020) 11:579362. doi: 10.3389/fmicb.2020.579362 33224118 PMC7669749

[35] Sun Y Gao L Yuan M Yuan L Yang J Zeng T. *In vitro* and *in vivo* Study of Antifungal Effect of Pyrvinium Pamoate Alone and in Combination With Azoles Against Exophiala dermatitidis. Front Cell Infect Microbiol. (2020) 10:576975. doi: 10.3389/fcimb.2020.576975 33194816 PMC7649562

[36] Downey AS Chong CR Graczyk TK Sullivan DJ. Efficacy of pyrvinium pamoate against Cryptosporidium parvum infection *in vitro* and in a neonatal mouse model. Antimicrob Agents Chemother. (2008) 52:3106–12. doi: 10.1128/AAC.00207-08 PMC2533469 18591280

[37] Zhang X Li B Huo S Du J Zhang J Song M. Hexafluoropropylene oxide trimer acid exposure triggers necroptosis and inflammation through the Wnt/beta-catenin/NF-kappaB axis in the liver. Sci Total Environ. (2023) 905:167033. doi: 10.1016/j.scitotenv.2023.167033 37709082

[38] Liu J Xiang J Li X Blankson S Zhao S Cai J. NF-κB activation is critical for bacterial lipoprotein tolerance-enhanced bactericidal activity in macrophages during microbial infection. Sci Rep. (2017) 7:40418. doi: 10.1038/srep40418 28079153 PMC5227741

[39] Chai Q Yu S Zhong Y Lu Z Qiu C Yu Y. A bacterial phospholipid phosphatase inhibits host pyroptosis by hijacking ubiquitin. Science. (2022) 378:eabq0132. doi: 10.1126/science.abq0132 36227980

[40] Rastogi S Ellinwood S Augenstreich J Mayer-Barber KD Briken V. Mycobacterium tuberculosis inhibits the NLRP3 inflammasome activation via its phosphokinase PknF. PloS Pathog. (2021) 17:e1009712. doi: 10.1371/journal.ppat.1009712 34324582 PMC8321130

[41] Yang J Ma Y Yu J Liu Y Xia J Kong X. Advancing roles and therapeutic potentials of pyroptosis in host immune defenses against tuberculosis. Biomolecules. (2024) 14(10):1255. doi: 10.3390/biom14101255 39456188 PMC11505957

[42] Nisa A Kipper FC Panigrahy D Tiwari S Kupz A Subbian S. Different modalities of host cell death and their impact on Mycobacterium tuberculosis infection. Am J Physiol Cell Physiol. (2022) 323:C1444–c74. doi: 10.1152/ajpcell.00246.2022 PMC9662802 36189975

[43] Vu A Glassman I Campbell G Yeganyan S Nguyen J Shin A. Host Cell Death and Modulation of Immune Response against Mycobacterium tuberculosis Infection. Int J Mol Sci. (2024) 25(11):6255. doi: 10.3390/ijms25116255 38892443 PMC11172987

[44] Man SM Karki R Kanneganti TD. Molecular mechanisms and functions of pyroptosis, inflammatory caspases and inflammasomes in infectious diseases. Immunol Rev. (2017) 277:61–75. doi: 10.1111/imr.12534 28462526 PMC5416822

[45] Steinhart Z Angers S. Wnt signaling in development and tissue homeostasis. Development. (2018) 145(11):dev146589. doi: 10.1242/dev.146589 29884654

[46] Ratajczak MZ Kucia M. SARS-CoV-2 infection and overactivation of Nlrp3 inflammasome as a trigger of cytokine "storm" and risk factor for damage of hematopoietic stem cells. Leukemia. (2020) 34:1726–9. doi: 10.1038/s41375-020-0887-9 PMC7262681 32483300

[47] Pan P Shen M Yu Z Ge W Chen K Tian M. SARS-CoV-2 N protein promotes NLRP3 inflammasome activation to induce hyperinflammation. Nat Commun. (2021) 12:4664. doi: 10.1038/s41467-021-25015-6 34341353 PMC8329225

[48] Zeng J Xie X Feng XL Xu L Han JB Yu D. Specific inhibition of the NLRP3 inflammasome suppresses immune overactivation and alleviates COVID-19 like pathology in mice. EBioMedicine. (2022) 75:103803. doi: 10.1016/j.ebiom.2021.103803 34979342 PMC8719059

[49] Obare LM Temu T Mallal SA Wanjalla CN. Inflammation in HIV and its impact on atherosclerotic cardiovascular disease. Circ Res. (2024) 134:1515–45. doi: 10.1161/circresaha.124.323891 PMC11122788 38781301

[50] Minns MS Liboro K Lima TS Abbondante S Miller BA Marshall ME. NLRP3 selectively drives IL-1β secretion by Pseudomonas aeruginosa infected neutrophils and regulates corneal disease severity. Nat Commun. (2023) 14:5832. doi: 10.1038/s41467-023-41391-7 37730693 PMC10511713

[51] Gao Y Yu S Chen M Wang X Pan L Wei B. cFLIP(S) regulates alternative NLRP3 inflammasome activation in human monocytes. Cell Mol Immunol. (2023) 20:1203–15. doi: 10.1038/s41423-023-01077-y PMC10541859 37591930

[52] Lee J Sasaki F Koike E Cho M Lee Y Dho SH. Gelsolin alleviates rheumatoid arthritis by negatively regulating NLRP3 inflammasome activation. Cell Death Differ. (2024) 31:1679–94. doi: 10.1038/s41418-024-01367-6 PMC11618363 39179640

[53] Leask MP Crişan TO Ji A Matsuo H Köttgen A Merriman TR. The pathogenesis of gout: molecular insights from genetic, epigenomic and transcriptomic studies. Nat Rev Rheumatol. (2024) 20:510–23. doi: 10.1038/s41584-024-01137-1 38992217

