# Supplementary material for: Suppression of NLRP3 inflammasome by a small molecule targeting CK1α–β-catenin–NF-κB and CK1α–NRF2–mitochondrial OXPHOS pathways during mycobacterial infection
Source: Front Immunol. 2025 Feb 28;16:1553093. doi: 10.3389/fimmu.2025.1553093 (PMC11906677; doi:10.3389/fimmu.2025.1553093)
Supplement: Supplementary file 1 [file DataSheet1.docx]

Supplementary Material

# Supplementary Figures and Tables

## Supplementary Figures


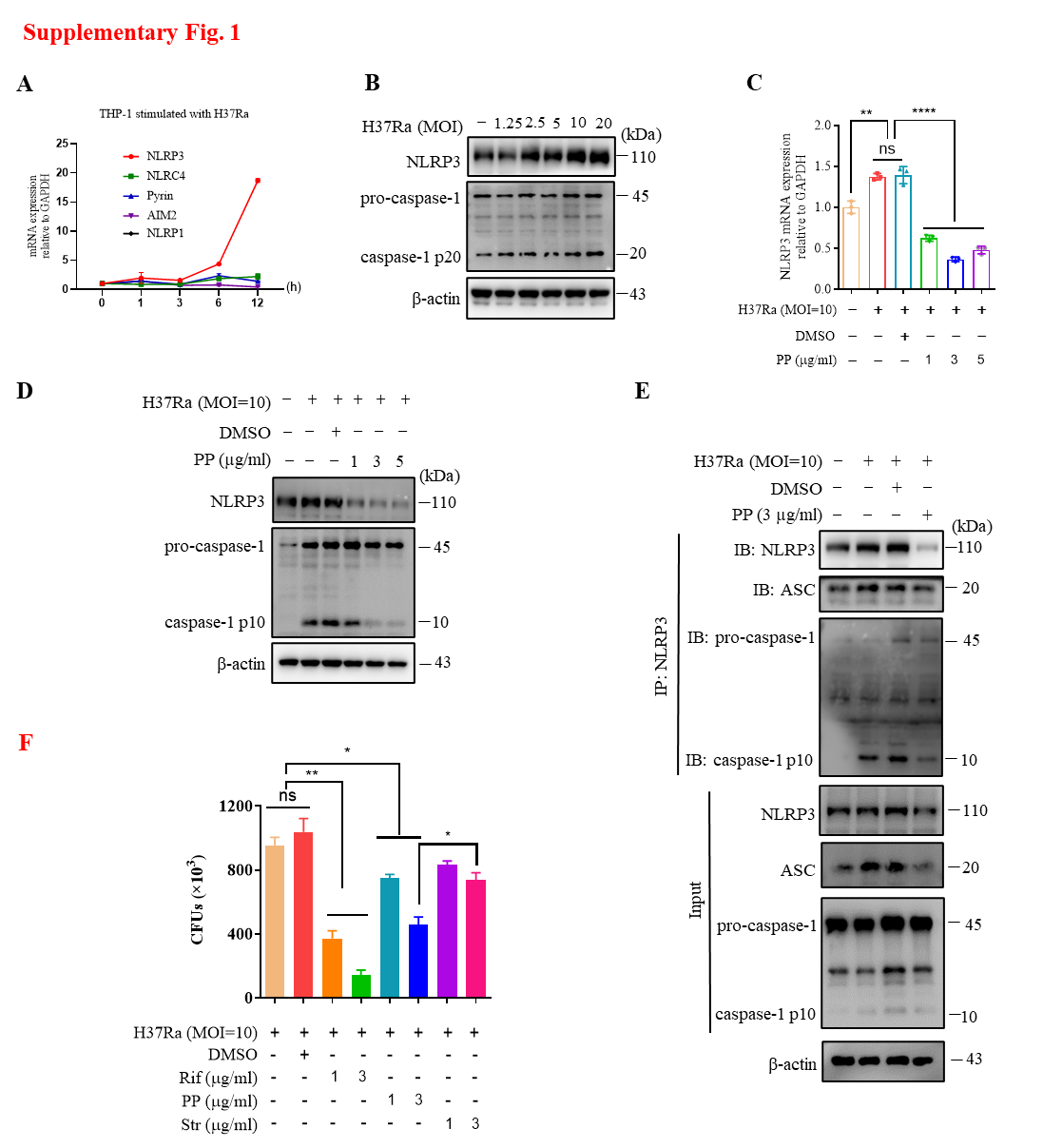


**Supplementary Fig. 1**. **PP suppresses NLRP3–ASC–Casp-1 inflammasome activation in *M.tb* H37Ra-infected THP-1 cells.** (A) RT-qPCR assay of the mRNA expression of NLRP1, NLRP3, NLRC4, pyrin and AIM2 in H37Ra-infected THP-1 cells at different time points. (B) WB assay of the protein expression of NLRP3 and caspase-1 in THP-1 cells. THP-1 cells were stimulated with H37Ra at different MOI for 4 h, and then cultured for another 24 h after washing with PBS for 3 times. (C, D) The mRNA and protein expression of NLRP3 or pro-caspase-1 by RT-qPCR (C) and WB assay (D) in THP-1 cells. THP-1 cells were infected with H37Ra (MOI=10) for 4 h and then treated with the indicated concentration of PP for another 24 h. (E) THP-1 cells infected with H37Ra (MOI=10) for 4 h, and then treated with PP for another 24 h were subjected to IP with anti-NLRP3 antibody and IB with antibodies against NLRP3, ASC, pro-caspase-1 and caspase-1 p10. (F) CFUs of *M.tb* H37Ra in THP-1 cells after PP treatment. THP-1 cells were infected with H37Ra (MOI=10) for 4 h and then treated with PP, Str or Rif for 24 h. Str, streptomycin; Rif, Rifampicin. Data represent mean ± SD for three independent experiments. One-way ANOVA followed by Tukey’s multiple comparison test was used to assess the statistical difference for C and F.


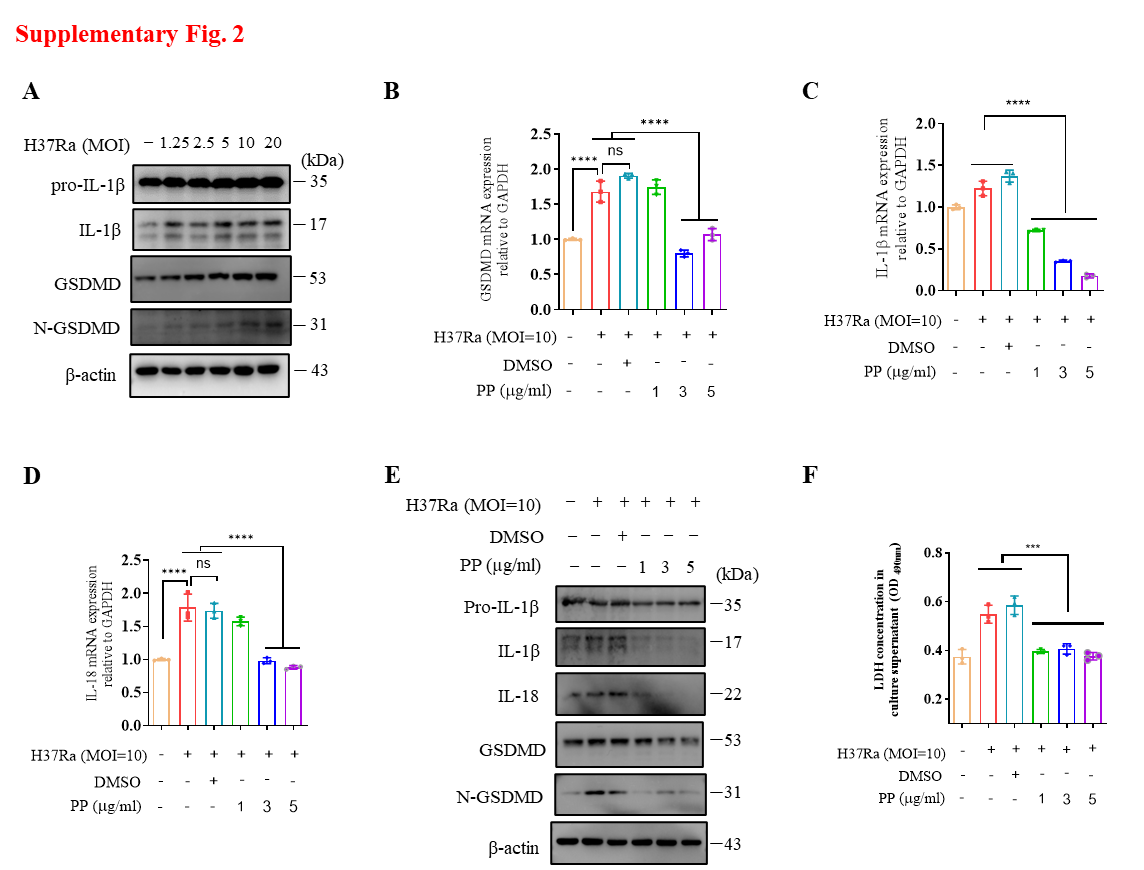


**Supplementary Fig. 2**. **PP suppresses *M.tb* H37Ra-induced GSDMD cleavage and IL-1β production in THP-1 cells**. (A) WB assay of pro-IL-1β, IL-1β, GSDMD and N-GSDMD protein expression in THP-1 cells. THP-1 cells were stimulated with H37Ra at different MOI for 4 h, and then cultured for another 24 h after washing with PBS for 3 times. (B-D) RT-qPCR assay of GSDMD (B), IL-1β (C) and IL-18 (D) mRNA expression in THP-1 cells. (E) WB assay of pro-IL-1β, IL-1β, IL-18, GSDMD and N-GSDMD protein expression in THP-1 cells. (F) LDH assay of cell viability in THP-1 cells. The THP-1 cells were infected with H37Ra (MOI=10) for 4 h and then treated with different concentrations of PP for another 24 h. Data represent mean ± SD for three independent experiments. One-way ANOVA followed by Tukey’s multiple comparison test was used to assess the statistical difference for B-D, F.

**
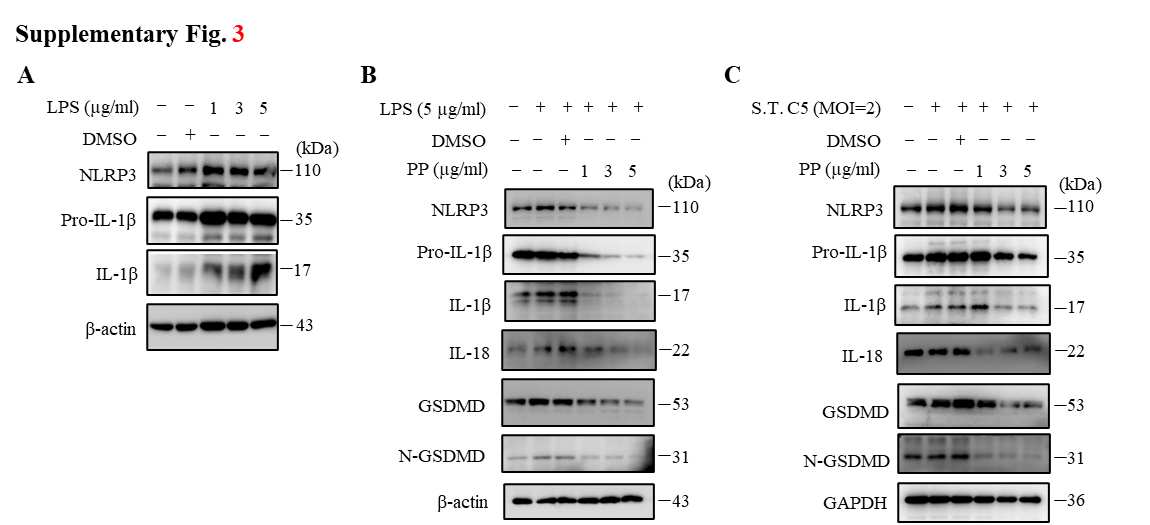
**

**Supplementary Fig. 3**. **PP suppresses NLRP3–GSDMD–pro-IL-1β expression in LPS-treated or *S. typhimurium* C5-infected THP-1 cells**. (A) WB assay of NLRP3, pro-IL-1β and IL-1β protein expression in THP-1 cells. THP-1 cells were stimulated with different concentrations of LPS. (B) WB assay of NLRP3, pro-IL-1β, IL-18, GSDMD and N-GSDMD protein expression in THP-1 cells. THP-1 cells were stimulated with LPS (5 µg/ml) for 4 h and then treated with different concentrations of PP for another 24 h. (C) WB assay of NLRP3, pro-IL-1β, IL-18, GSDMD and N-GSDMD protein expression in THP-1 cells. THP-1 cells were infected with *Salmonella typhimurium* C5 (MOI=2) for 4 h and then treated with different concentrations of PP for another 24 h.

**
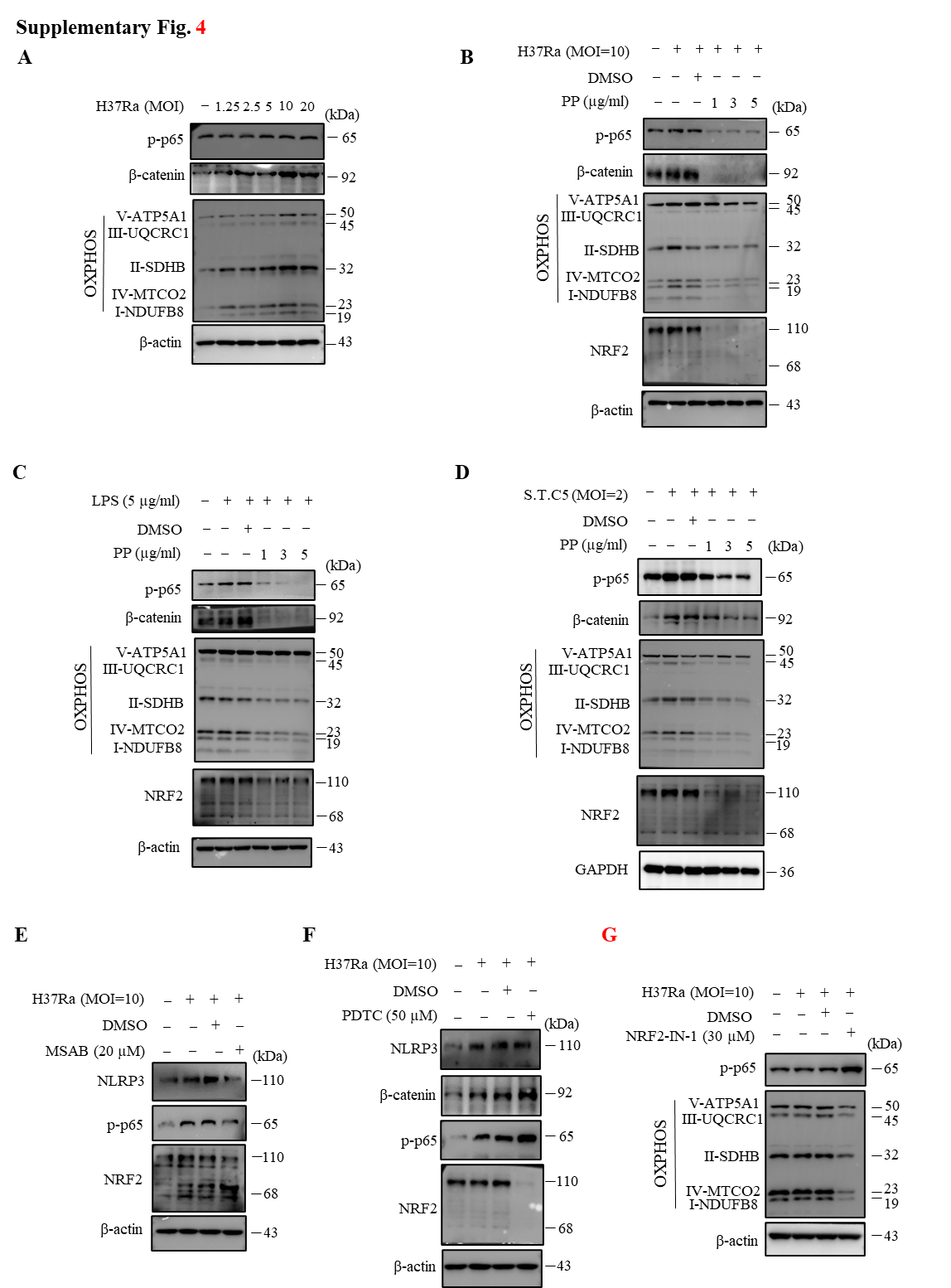
**

**Supplementary Fig. 4**. **PP suppresses H37Ra, LPS or *S. typhimurium C5* infection-induced NLRP3 activation via repressing the β-catenin–NF-κB signaling pathway and mitochondrial oxidative phosphorylation (OXPHOS)**. (A) WB assay of p-p65, β-catenin, mitochondrial OXPHOS-related protein expression in THP-1 cells. THP-1 cells were stimulated with H37Ra at different MOI for 4 h, and then cultured for another 24 h after washing with PBS for 3 times. (B) WB assay of p-p65, β-catenin, mitochondrial OXPHOS-related protein and NRF2 protein expression in THP-1 cells. THP-1 cells were infected with H37Ra (MOI=10) for 4 h and then treated with different concentrations of PP for another 24 h. (C) WB assay of p-p65, β-catenin, mitochondrial OXPHOS-related proteins and NRF2 protein expression in THP-1 cells. THP-1 cells were stimulated with LPS (5 µg/ml) for 4 h and then treated with different concentrations of PP for another 24 h. (D) WB assay of p-p65, β-catenin, mitochondrial OXPHOS-related proteins and NRF2 protein expression in THP-1 cells. THP-1 cells were infected with *S. typhimurium* C5 (S.T.C5, MOI=2) for 4 h and then treated with different concentrations of PP for another 24 h. (E) WB assay of NLRP3, p-p65 and NRF2 protein expression in THP-1 cells. THP-1 cells were infected with H37Ra (MOI=10) for 4 h and then treated with MSAB (β-catenin inhibitor) at 20 µM for another 24 h. (F) WB assay of NLRP3, β-catenin, p-p65 and NRF2 protein expression in THP-1 cells. THP-1 cells were infected with H37Ra (MOI=10) for 4 h and then treated with PDTC (NF-κB inhibitor) at 50 µM for another 24 h. (G) WB assay of p-p65 and OXPHOS-related protein expression in THP-1 cells. THP-1 cells were infected with H37Ra (MOI=10) for 4 h and then treated with NRF2-IN-1 (NRF2 inhibitor) at 30 µM for another 24 h.


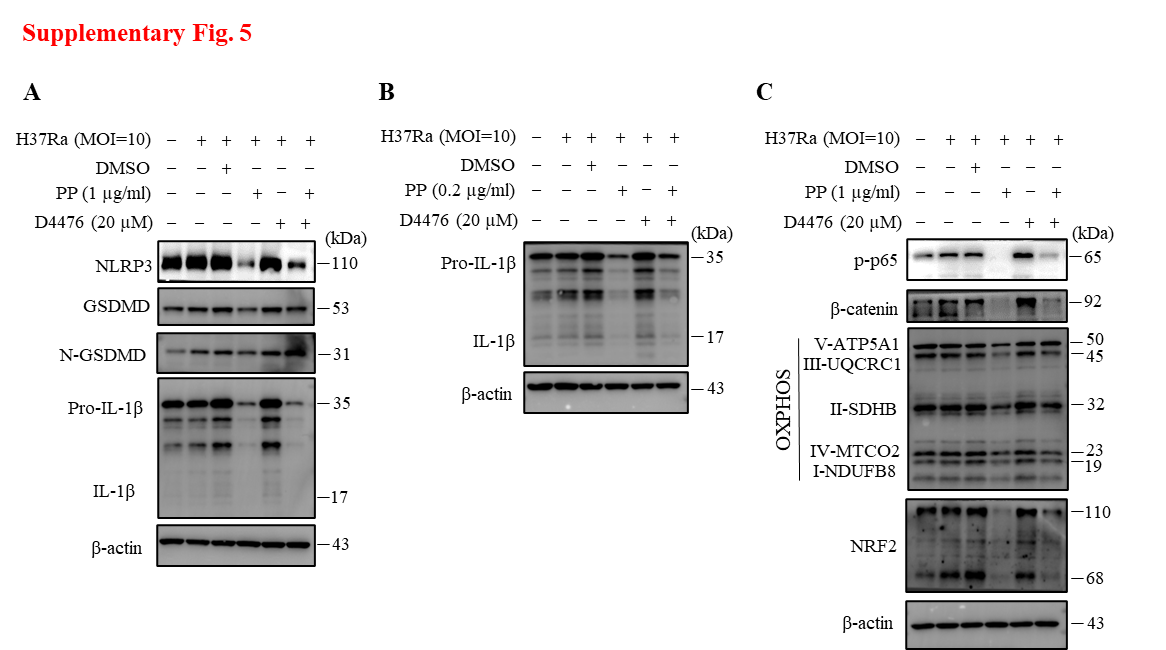


**Supplementary Fig. 5. CK1α specific inhibitor D4476 reverses the inhibitory effects of PP on H37Ra-induced inflammatory responses in macrophages**. (A) WB assay of NLRP3, GSDMD and IL-1β protein expression in THP-1 cells. THP-1 cells were infected with H37Ra (MOI=10) for 4 h and then treated with PP and/or D4476 (CK1α inhibitor) at 20 µM for another 24 h. (B) WB assay of pro-IL-1β and IL-1β protein expression in H37Ra-infected THP-1 cells that were treated with/without PP and/or D4476. (C) WB assay of p-p65, CK1α, β-catenin, NRF2 and mitochondrial OXPHOS-related protein expression in H37Ra-infected THP-1 cells that were treated with/without PP and/or D4476.

## Supplementary Tables

Supplementary Table 1. The primers used for RT-qPCR.

| **Primer name** | | **Primer sequence** |
| --- | --- | --- |
| NLRP1 | Forward | 5′- CCACAACCCTCTGTCTACATTAC -3′ |
|  | Reverse | 5′- GCCCCATCTAACCCATGCTTC -3′ |
| NLRP3 | Forward | 5′- CCCCGTGAGTCCCATTA -3′ |
|  | Reverse | 5′- GACGCCCAGTCCAACAT -3′ |
| NLRC4 | Forward | 5′- CCAGTCCCCTCACCATAGAAG -3′ |
|  | Reverse | 5′- ACCCAAGCTGTCAGTCAGACC -3′ |
| AIM2 | Forward | 5′- CTGCAGTGATGAAGACCATTCGTA -3′ |
|  | Reverse | 5′- GGTGCAGCACGTTGCTTTG -3′ |
| pyrin | Forward | 5′-TCATTTTCCCTCAGAACCCC-3′ |
|  | Reverse | 5′-CAATCCAGTCTGCTTGCGTT-3′ |
| pro-IL-1β | Forward | 5′- CCTGTGGCCTTGGGCCTCAA -3′ |
|  | Reverse | 5′- GGTGCTGATGTACCAGTTGGG -3′ |
| GSDMD | Forward | 5′-GTGTGTCAACCTGTCTATCAAGG-3′ |
|  | Reverse | 5′-CATGGCATCGTAGAAGTGGAAG-3′ |
| IL-18 | Forward | 5′-CTGCCACCTGCTGCAGTCTA-3′ |
|  | Reverse | 5′-TCTACTGGTTCAGCAGCCATCTTTA-3′ |
| GAPDH | Forward | 5′- ACCACAGTCCATGCCATCAC-3′ |
|  | Reverse | 5′- TCCACCACCCTGTTGCTGTA-3′ |
| MT-ND1 | Forward | 5′-CCACCTCTAGCCTAGCCGTTTA-3′ |
|  | Reverse | 5′-GGGTCATGATGGCAGGAGTAAT-3′ |
| MT-ND2 | Forward | 5′- ACCAAATCTCTCCCTCACTAAACG-3′ |
|  | Reverse | 5′-CCACCTCAACTGCCTGCTATG -3′ |
| MT-ND3 | Forward | 5′- CCCTTACGAGTGCGGCTTC-3′ |
|  | Reverse | 5′-AGTGGCAGGTTAGTTGTTTGTAGG -3′ |
| MT-ND4 | Forward | 5′-AGCCCTCGTAGTAACAGCCATTC -3′ |
|  | Reverse | 5′- AGTGCGTTCGTAGTTTGAGTTTGC-3′ |
| MT-ND4L | Forward | 5′-ACCTCACCATAGCCTTCTCAC -3′ |
|  | Reverse | 5′-TAGTCCTACAGCTGCTTCGC -3′ |
| MT-ND5 | Forward | 5′-ATCGGCTGAGAGGGCGTAGG -3′ |
|  | Reverse | 5′-GCTTGAATGGCTGCTGTGTTGG -3′ |
| MT-ND6 | Forward | 5′- GTGTGGTCGGGTGTGTTATTATTC-3′ |
|  | Reverse | 5′-CAATCCTACCTCCATCGCTAACC-3′ |
| MT-CO1 | Forward | 5′-GCAGGAACAGGTTGAACAGTCTAC-3′ |
|  | Reverse | 5′-GGGCGTTTGGTATTGGGTTATGG-3′ |
| MT-CO2 | Forward | 5′-GCGACCTGCGACTCCTTGAC-3′ |
|  | Reverse | 5′-GTGTAGCGGTGAAAGTGGTTTGG-3′ |
| MT-CO3 | Forward | 5′-CCACAGGCTTCCACGGACTTC-3′ |
|  | Reverse | 5′-GTATCAGGCGGCGGCTTCG-3′ |
| MT-CYB | Forward | 5′- GCGTCCTTGCCCTATTACTATCC-3′ |
|  | Reverse | 5′-GCTTACTGGTTGTCCTCCGATTC-3′ |
| MT-ATP6 | Forward | 5′-CGGGCACAGTGATTATAGGCTTTC-3′ |
|  | Reverse | 5′-TTGGTTGAATGAGTAGGCTGATGG-3′ |
| MT-ATP 8 | Forward | 5′-ACAGTGAAATGCCCCAACTAAAT-3′ |
|  | Reverse | 5′-AGGGAGGTAGGTGGTAGTTTGTG-3′ |
| NFE2L2 (NRF2) | Forward | 5′-TATCCATTCCTGAGTTACAGTGTC-3′ |
|  | Reverse | 5′-CTGTCAGTTTGGCTTCTGGAC-3′ |

**Supplementary Table 2. The sequences of CK1α siRNA**

| **CK1α siRNA** | **Oligo sequence** |
| --- | --- |
| CK1α siRNA-1 | 5’-UUCAUUGUCGGAGGGAAAUTT-3’ |
|  | 5’-AUUUCCCUCCGACAAUGAATT-3’ |
| CK1α siRNA-2 | 5’-CUGUUCAAGAAGGUUCACATT-3’ |
|  | 5’-UGUGAACCUUCUUGAACAGTT-3’ |
| CK1α siRNA-3 | 5’-UGCAGAAUUUGCGAUGUACTT-3’ |
|  | 5’-GUACAUCGCAAAUUCUGCATT-3’ |

Supplementary Note

The raw RNA-seq data generated in this study can be found in the Genome Sequence Archive for Human (1) at the National Genomics Data Center, China National Center for Bioinformation / Chinese Academy of Sciences (https://ngdc.cncb.ac.cn/, Accession No.: CRA021722).

**Supplementary References**

1. Xue YB, Bao YM, Zhang Z, Zhao WM, Xiao JF, He SM, et al. Database Resources of the National Genomics Data Center, China National Center for Bioinformation in 2022. *Nucleic Acids Res* (2022) 50:D27-D38. doi: 10.1093/nar/gkab951.
